# Latent profiles of movement behaviour compositions and their associations with adiposity and health-related quality of life in Australian children: a cross-sectional and 12-month longitudinal study

**DOI:** 10.1136/bmjopen-2025-109130

**Published:** 2026-06-08

**Authors:** Simone JJM Verswijveren, Aaron Miatke, Kylie D Hesketh, Nicola D Ridgers, Ana María Contardo Ayala,, Dorothea Dumuid, Anna Timperio, Charlotte Lund Rasmussen, Ty E Stanford, Narcis Gusi, Jo Salmon

**Affiliations:** 1 Institute for Physical Activity and Nutrition (IPAN), School of Exercise and Nutrition Sciences, Deakin University, Geelong, Victoria, Australia; 2 Alliance for Research in Exercise, Nutrition and Activity (ARENA), Adelaide University, Adelaide, South Australia, Australia; 3 Centre for Adolescent Health, Murdoch children’s Research Institute, Parkville, Victoria, Australia; 4 Murdoch children’s Research Institute, Parkville, Victoria, Australia; 5 Curtin School of Allied Health, Faculty of Health Sciences, Curtin University, Perth, Western Australia, Australia; 6 Faculty of Sport Sciences, University of Extremadura, Cáceres, Spain

**Keywords:** Child, PUBLIC HEALTH, EPIDEMIOLOGY

## Abstract

**Objectives:**

To identify profiles of compositional movement behaviour patterns among children and examine cross-sectional and 12-month associations with adiposity markers and health-related quality of life (HRQoL).

**Design:**

Secondary analysis of data from the TransformUs cluster randomised controlled trial with cross-sectional and 12-month follow-up analyses.

**Setting:**

Primary schools in metropolitan and regional areas of Victoria, Australia.

**Participants:**

Children aged 7–11 years with valid accelerometer at baseline, regardless of demographic, adiposity and HRQoL data available (n=792), were included in the analytical sample for the latent profile analysis.

**Measures:**

Sedentary time, light-intensity physical activity (LPA) and moderate- to vigorous-intensity physical activity (MVPA) along with their respective mean bout lengths were derived from raw acceleration data. Latent profile analysis used these measures (total times, as isometric log ratios and mean bout lengths) as input variables to classify distinct profiles for us as a categorical exposure variable in regression models. Primary outcomes were age- and sex-standardised body mass index, waist circumference and parent-reported HRQoL at baseline. Secondary outcomes were the same measures assessed at 12-month follow-up.

**Results:**

Four distinct profiles were identified. The *high MVPA-short sedentary bout* profile (n=184) was characterised by the highest levels of MVPA, moderate sedentary time and the shortest mean sedentary bout duration. The *low sedentary-high LPA* profile (n=54) had the lowest sedentary time, the highest LPA and the longest mean LPA bout duration. Two profiles were characterised by high sedentary time: the *high sedentary-long sedentary bout* profile (n=149), which had the longest mean sedentary bout durations, and the *high sedentary-shorter bouts* profile (n=405), which also had high sedentary time but shorter bout durations for all intensities. While *the omnibus Wald test for differences across profiles indicated uncertainty in the overall profile effect, the high MVPA-short sedentary bout* profile had favourable adiposity levels cross-sectionally compared with the *high sedentary-long sedentary bout* reference profile in pairwise comparisons. No longitudinal associations were detected.

**Conclusions:**

Four distinct movement profiles were identified. Few pairwise differences between health outcomes were observed. While MVPA remains a key factor for promoting healthy body weight, our findings suggest that a variety of movement patterns - including those characterised by lower sedentary time and higher LPA - may also support health in children.

**Trial registration:**

This study is a secondary analysis of the TransformUs effectiveness-implementation trial, registered with the Australian Clinical Trials Registry (ACTRN12617000204347; 1 April 2017).

**STRENGTHS AND LIMITATIONS OF THIS STUDY**

- This study is particularly novel for its application of a combination of compositional analysis and latent profile analysis, which not only examined total movement behaviours but also how these behaviours were accumulated (ie, through shorter or longer mean bout durations).
- The longitudinal design, combined with device-based measurement of movement behaviours, enabled assessment of both cross-sectional and 12-month associations with health outcomes.
- A limitation of this study is that it did not account for sleep behaviours or diet, which are known to influence physical activity patterns and health outcomes in children.
- Only 57.8% of consented participants provided valid accelerometer data, which may have introduced selection bias.
- The relatively small size of one latent profile and data collection during the COVID-19 pandemic may limit generalisability of findings.

## Background

 The time children spend sitting and being physically active (collectively called ‘waking movement behaviours’) is critical for their short- and long-term health, including cardio-metabolic health and well-being.^1 2^ Consequently, organisations including the WHO and the Australian Government advocate for regular physical activity and reducing sedentary behaviours across all ages.^3 4^ Both recommend 5–17-year-olds accumulate ≥60 min of moderate- to vigorous-intensity physical activity (MVPA), break up extended periods of sitting and limit recreational sedentary screen time to <2 hours per day. However, only approximately 27%–30% and 34%–39% of children aged 5–17 globally meet these MVPA and screen time recommendations, respectively.^5^

Movement behaviour guidelines recognise the importance of sufficient physical activity across a range of intensities and limiting sedentary behaviour for children’s health.^6^ However, waking movement behaviours can be accumulated in many ways. However, waking movement behaviours can be accumulated in different ways. These movement behaviour patterns reflect the temporal structure of physical activity and sedentary behaviour during waking hours and are commonly operationalised as activity and sedentary bouts.^7^ Evidence regarding which patterns are most beneficial for health remains inconsistent (eg, in children aged 7–13-year-olds^8 9^ and 6–19-year-olds^10^), and existing research is largely cross-sectional,^1 2 11^ limiting understanding of the longer-term health implications of suboptimal activity patterns in childhood. Longitudinal studies that move beyond total volumes to examine activity patterns are therefore needed to better inform interventions and policy aimed at improving children’s health and wellbeing.

It is important to move beyond analyses of individual waking movement behaviours towards integrated approaches such as compositional data analysis (CoDA), which accounts for the co-dependency of movement behaviours and enables simultaneous consideration of all activity intensities across the day. Previous CoDA studies have linked school-aged children’s movement behaviour compositions to adiposity^12 13^ and health-related quality of life (HRQoL).^11^ However, most existing work has applied CoDA within *variable-centred* approaches (eg, time reallocation regression models) that assume population homogeneity, thereby overlooking distinct subgroups with different activity accumulation patterns. In contrast, *person-centred* approaches such as latent profile analysis allow identification of multiple behavioural profiles within a population and capture meaningful differences in both their movement compositions and consequent health outcomes.^14^ Integrating CoDA with latent profile analysis is therefore essential to accurately capture and characterise real-world movement behaviour patterns and their health implications in children.

While a small number of studies have used the combination of CoDA and latent profile analyses to assess movement behaviours and detect associations with adiposity markers,^15 16^ these were mostly cross-sectional, focused on older age groups and did not include associations with other health markers such as HRQoL. Further, none went beyond broad measures of daily activity time to generate profiles of accumulation of activity patterns, such as mean bout durations.

The aim of this study was to use CoDA in conjunction with latent profile analysis to identify profiles of children based on distinct activity accumulation patterns and investigate cross-sectional and 12-month associations between these profiles and adiposity markers and HRQoL.

## Methods

### Data source

Analyses were conducted using data from the TransformUs hybrid effectiveness-implementation trial (protocol paper included as online supplementary file 1).^17^ TransformUs (https://transformus.com.au) is an efficacious^18^ whole-of-school programme delivered by teachers to support children’s movement behaviours by focusing on reducing and breaking up sitting and increasing physical activity throughout the school day. It was implemented at scale in Victorian primary schools (a state in south-eastern Australia). Schools in New South Wales (NSW; an adjacent Australian state) were included as usual practice controls. The current study used baseline and 12-month effectiveness data only. Further information can be found in the published protocol paper.^17^ Participants gave informed consent to participate in the study before taking part. School principals were provided with a plain language statement and consent form to sign for their school to participate. All children in years 3 and 4 (aged 7–11 years) at consenting schools were invited to participate, and written parental consent and verbal child assent were obtained prior to data collection (n=1370 children (n=788 in Victoria, n=582 in NSW)). Reporting in the present manuscript follows the Strengthening the Reporting of Observational Studies in Epidemiology (STROBE) statement (Online supplementary file 2, Table S1).^19^

### Recruitment

For the effectiveness trial, 21 Victorian schools of varying socioeconomic status (SES) and geographical areas were invited to participate (2019). 19 control schools in NSW, Australia, were then recruited in a targeted manner to match the Victorian schools with regard to school size, type, SES and geographical location.

### Data collection and measures

Movement behaviour (via accelerometers) and health outcomes (adiposity and HRQoL) were collected at baseline (June 2018–October 2019) and 12-month follow-up (June 2019–December 2020). All measurement protocols were identical at both time points; however, the COVID-19 pandemic impacted data collection protocols during 2020,^17^ which meant that some students due for follow-up that year could not be assessed in school as originally planned. Instead, surveys were completed online, and anthropometric measurements were not collected for those children. This led to missing anthropometry data at follow-up for a subset of the cohort, reducing the available sample size for longitudinal analyses of adiposity outcomes and potentially limiting statistical power and representativeness. Baseline demographic variables were collected through parent survey.

#### Accelerometer-derived movement behaviours

Of the 1370 consenting students, 1296 (94.6%) participated in baseline data collection, of whom 1197 (92.4%) were provided with an accelerometer to wear on their right hip for a period of 8 days. The ActiGraph (model GT3X) devices were set up with a sample frequency of 30 Hz and normal filter. The ActiGraph is the most widely used accelerometer and has the strongest evidence base to support its feasibility, reliability and validity in studies of school-aged children and adolescents^20^ with 73.7%–96.0% balanced accuracy for detecting sedentary time and physical activity.^21^ Participants were instructed to wear the device for all waking hours and only to remove the device while sleeping or during water-based activities (eg, bathing, swimming, etc). ActiGraph data were downloaded using ActiLife V.6.11.4 and saved in raw .gt3x file format for data processing using GGIR^22^ http://cran.r-project.org From the processed accelerometer data, daily variables were created for sedentary time, light-intensity physical activity (LPA), moderate-to-vigorous physical activity (MVPA; derived as the sum of moderate- and vigorous-intensity activity) and associated duration-based metrics, as described in online supplementary file 2, table S2).

#### Adiposity markers

Height, weight and waist circumference (WC) data were systematically collected in schools at both baseline and follow-up by trained research assistants. Height was assessed using portable stadiometers (SECA 220, Los Angeles, California, USA) and weight was assessed using digital scales (Wedderburn Tanita, Melbourne, Victoria, Australia).^17^ Consequently, continuous WHO Child Growth Standards age- and sex-standardised z-scores (zBMI) were computed based on BMI (kg/m²).^23^ Children were subsequently classified as underweight (zBMI<−2), healthy-weight (≥−2; ≤+1), overweight (>+1; ≤2) or obese (>+2) based on calculated z-scores. WC was assessed using a flexible steel tape at the narrowest point between the bottom rib and the iliac crest in the midaxillary plane.^17^ Australian percentile curves for WC were then used to determine continuous age-specific and sex-specific WC standardised z-scores (zWC) as well as WC categories for healthy-weight (<75%), overweight (75%–90%) and obese (>90%).^24^

#### Self-reported HRQoL and perceived overall health

Baseline HRQoL and overall health data were obtained through a survey with five items from the EQ-5D-Y-3L questionnaire, a child-friendly self-complete instrument developed by the EuroQol Research Foundation for use by children aged 8–15 years; TransformUs study registration ID on the EuroQol website: 52308). The EQ-5D-Y-3L is validated for use in 8–15-year-olds via self-report.^25^ The five categorical items assessed specific dimensions: (1) mobility, (2) independence, (3) usual activities, (4) pain/discomfort and (5) feelings of being worried or sad. Each dimension had three response levels—for example, no problems, some problems and a lot of problems. A continuous HRQoL score was calculated based on Australian preference-value sets.^26^ The survey also included one Visual Analogue Scale (VAS) item for perceived overall health: ‘How is your health today?’ which ranged from 0 (indicating worst imaginable health) to 100 (reflecting best imaginable health).

#### Demographic characteristics

Parents reported their child’s date of birth, gender and postcode via consent and survey items. Four gender categories (girl, boy, other or I prefer not to say) were presented in the survey, however, no parents of children in the analysed sample selected ‘other’ or ‘I prefer not to say’ and therefore this variable was treated as binary. Area-level SES was derived from the home postcode using the Census of Population and Housing: Socio-Economic Indexes for Areas (SEIFA).^27^ Data were transformed into SES approximate tertiles based on SEIFA’s Index of Relative Socioeconomic Advantage and Disadvantage deciles: low (1st–3rd decile), mid (4th–7th decile) and high (8th–10 th decile).^27^ On inspection of the data, low and mid SES categories were merged as few participants (9.7%) resided in low SES areas. Geographical location was determined via school level classifications as reported by the Australian Curriculum, Assessment and Reporting Authority and based on remoteness area as calculated by the Australian Bureau of Statistics.^28^ All participants were classified as residing in either major city or inner regional, with none residing in outer regional or remote/very remote areas.

### Statistical analysis

Statistical analyses were conducted in RStudio Version 2023.06.2 (RV.4.3.1), using mclust::Mclust() function for the latent profile analysis. Participant demographic characteristics were summarised as arithmetic mean±SD for continuous variables or counts with percentages for categorical variables. Data from all participants with valid accelerometry wear at baseline (n=792; 57.8% of participants with consent) regardless of demographic and health data availability, were included in the analytical sample for the latent profile analysis. Participants included in the longitudinal analyses were compared with those with no follow-up data with either a Chi-squared test (categorical variables) or a one-way ANOVA (continuous variables). The significance level of alpha=0.05 was used for all analyses. The statistical analyses are presented consistent with the CHecklist for statistical Assessment of Medical Papers (online supplementary file 2, table S3)^29^

#### Creating movement behaviour compositions with CoDA

Movement behaviour compositions were expressed as compositional means by taking the geometric mean of the three behaviours and closing the composition to 760 min/day (average waking wear time).^30^ The three-part movement behaviour compositions were expressed as two isometric log ratio (*ilr*) coordinates (variables) consistent with CoDA principles.^31^ No zero values were present in the movement behaviour data; therefore, no zero-replacement procedures were required prior to CoDA.

#### Latent profile analysis

The two CoDA-derived continuous *ilr* variables, together with the three continuous mean bout duration variables (not log transformed) for sedentary time, LPA and MVPA, were used as inputs for the latent profile analysis (ie, five continuous input variables in total). Four latent profile analysis model variants (equal volume, equal shape and undefined orientation (EEI); equal volume, equal shape and equal orientation (EEE); varying volume, varying shape and undefined orientation (VVI); and varying volume, varying shape, varying orientation (VVV)) were considered.^32 33^ The model variants differ in the extent to which they constrain or allow variance and covariance parameters within and between latent profiles.^32 33^ For example, the EEE structure constrains profiles to have equal variances and covariance patterns, such that profiles differ mainly in their mean values, whereas the VVV structure allows variances and covariances to vary freely across profiles, permitting differences in both central tendency and dispersion.

The optimal number of latent profiles (k*) was chosen from latent class models with k=1 to k=9 latent profiles within each of the four model variants. The preferred model variant and chosen k* were selected using a combination of the goodness-of-fit statistics: the Bayesian information criteria (BIC), Akaike’s information criteria (AIC), Sawa’s Adjusted Bayesian Information Criterion (SABIC), Consistent Akaike Information Criterion (CAIC), Adjusted Weighted Evidence (AWE), Integrated Completed Likelihood (ICL), Entropy, and Bootstrapped Likelihood Ratio Test Statistics (BLRTS). Lower BIC, AIC, SABIC, CAIC and AWE values indicate a better model fit,^34^ while higher ICL values indicate a better model fit. Entropy ranges from 0 to 1, with a value closer to 1 indicating greater profile separation.^34^ The BLRTS method sequentially compares results from the k+1 class model compared with the k class model, via a bootstrapped p value to assess an improvement in fit with the inclusion of one additional latent profile; the optimal k* for the BLRTS method is chosen as the smallest k where the associated test between the k and k+1 profile models has a p value≥to the level of significance.^35^ In addition to the goodness-of-fit statistics, hurdle criteria of latent class size (all k* profiles need to represent at least 5% of the included^36^) and profile interpretability (eg, whether they represented meaningful and unique observed groups) were also used. Goodness-of-fit statistics were also calculated for solutions including log-transformed mean bout durations (instead of the original mean bout durations) to explore potential improvements in skewness, and these results were compared with the original model to evaluate any better or different classifications. Log-transformed mean bout durations were compared with original mean bout durations via unweighted Cohen’s Kappa. All authors were invited to a meeting to achieve consensus on the optimal solution, goodness-of-fit statistics and interpretations. Once the optimal number of latent profiles was selected, the participants were assigned to their most likely profile, created profiles were described, labelled, and used in further regression analyses as a categorical predictor in the subsequent linear models.

#### Descriptive statistics of profiles and associations with health outcomes

Descriptive statistics of the k* latent profiles based on movement behaviour compositions, (mean, SD) for continuous variables or count (percentage; %) for categorical variables) movement behaviour and demographic variables were used to describe profile similarities and differences. Differences between profiles for categorical variables (gender, SES, geographical location, intervention arm) were tested using a Chi-squared test. Differences between profiles for continuous variables (age) were tested using a one-way ANOVA.

Linear mixed-effect regression models were fit on the continuous outcome variables of adiposity markers, composite HRQoL score and perceived health separately at baseline and 12-month follow-up using the derived profiles as a fixed categorical explanatory variable. Random intercepts were used to account for nesting at the school level. Baseline models were adjusted for age, gender, SES, except for zBMI and zWC models (where the outcome was already standardised to sex- and age-specific values) which were all included as fixed effects in the model. The 12-month follow-up models were also adjusted for baseline health outcomes and intervention arm. Omnibus Wald tests assessed overall differences across profiles. Intervention participants were included along with control participants in 12-month follow-up models due to the scaled-up intervention not being effective in changing time spent in behaviours between baseline and 12-month follow-up (data not yet published). Only participants with complete data for all exposure, dependent and covariate variables were included in these analyses (n=767 to n=776 (96.8–98.0% of participants with valid accelerometry wear at baseline), depending on outcome at baseline; n=437 to n=555 (55.2–70.1%), depending on outcome at follow-up).

Composite HRQoL and perceived health VAS scores were negatively skewed and censored at ‘perfect’ health. A mixed Tobit model^37^ was explored to fit these outcomes; however, the fit did not improve when compared with a standard linear mixed model. Thus, the linear mixed model was conducted. Sensitivity analyses were conducted for adiposity outcomes with the exclusion of underweight participants. In addition, a separate sensitivity analysis for longitudinal models only was conducted excluding participants from intervention schools at follow-up. Assumptions for the regression models were deemed plausible as assessed in diagnostic plots.

### Patient and public involvement

Patient or members of the public were not involved in the design or conduct of this current study. As described in the protocol paper,^17^ working closely with local councils and teachers, the TransformUs programme was adapted from the original Randomised control trial (RCT) design^18^ and tested in two pilot trials. Dissemination and implementation of the hybrid trial was conducted in partnership with the Victorian State Department of Education and six non-government organisations responsible for school education and teacher professional learning.

## Results

### Participant baseline characteristics

Participant characteristics are displayed in table 1. Nearly all (99%) of the 792 participants with valid accelerometry data provided adiposity and HRQoL data at baseline. At 12-month follow-up, 451 (55%) and 573 (72%) children provided adiposity and HRQoL data, respectively, with a mean follow-up time of 54 weeks (SD=3.9 weeks). Participants had a mean age of 9.1 years at baseline (SD=0.7) and approximately half of them were girls (51.1%). The mean zBMI was 0.5 (SD=1.1), with 29.3% classified as either overweight or obese. Average sedentary time was 582.5 min/day (SD=126), with LPA at 144.1 min/day (SD=74.9) and MVPA at 46.2 min/day (SD=18.5). Participants included in the longitudinal analyses were significantly more likely to be from a major city, older and have higher baseline zWC (all p<0.05). No differences were observed for sex, SES, profile membership, or baseline zBMI, QoL or VAS health.

**Table 1:** Participant characteristics

Participant numbers	
Original consented sample (n)	1370
Participated in baseline data collection (n)	1296
Analytical dataset with valid accelerometry at baseline (n)	792
Baseline demographic characteristics	
Age (years; mean (SD)	9.1 (0.7)
Gender (n (%)	
Girls	405 (51.1)
Boys	387 (48.9)
Socioeconomic status (n (%)	
Low	77 (9.9)
Mid	252 (32.4)
High	448 (57.7)
Intervention arm (n (%)	
VIC intervention group	493 (62.2)
NSW control group	299 (37.8)
Geographical location (n (%)	
Major city	592 (74.7)
Inner regional	200 (25.3)
Baseline health outcomes	
zBMI (mean±SD)^*^	0.5 (1.1)
BMI status (n (%)	
Underweight	7 (0.9)
Healthy weight	548 (69.9)
Overweight	147 (18.8)
Obese	82 (10.5)
zWC (mean (SD))^*^	0.6 (1.1)
WC status (n (%))	
Healthy weight	429 (54.2)
Overweight	160 (20.2)
Obese	203 (25.6)
VAS health score (mean (SD); range 0–100)	82.5 (18.4)
HRQoL composite score (mean (SD)	0.89 (0.13)
Mobility (n (%)	
No problems	709 (89.5)
Some problems	77 (9.7)
A lot of problems	6 (0.8)
Independence (n (%)	
No problems	720 (91.1)
Some problems	63 (8.0)
A lot of problems	7 (0.9)
Usual activity (n (%)	
No problems	671 (84.9)
Some problems	112 (14.2)
A lot of problems	7 (0.9)
Pain or discomfort (n (%)	
No pain or discomfort	414 (52.4)
Some pain or discomfort	343 (43.4)
A lot of pain or discomfort	33 (4.2)
Feelings (n (%))	
Not worried, sad or unhappy	554 (70.0)
A bit worried, sad or unhappy	214 (27.1)
Very worried, sad or unhappy	23 (2.9)
Baseline accelerometry variables^†^	
SED time (min/day, compositional mean)	579
LPA (min/day, compositional mean)	137
MVPA (min/day, compositional mean)	44
Mean duration SED bouts (min, mean (SD))	15.2 (6.1)
Mean duration LPA bouts (min, mean (SD))	2.8 (2.3)
Mean duration MVPA bouts (min, mean (SD))	2.4 (0.5)

Participants in the analytical sample for inclusion in the latent profile analysis had valid accelerometry wear at baseline (n=792; 57.8% of participants with parental consent) regardless of demographic and health data availability

* Continuous BMI and WC were transformed to age- and sex-standardised z-scores for use in analysis.

† Compositional means are calculated using log-ratio transformations that account for the co-dependent nature of time-use data. As a result, they may differ from arithmetic means, which are calculated independently for each behaviour.

BMI, body mass index; HRQoL, health-related quality of life; LPA, light-intensity physical activity; MVPA, moderate- to vigorous-intensity physical activity; NSW, New South Wales; SED, sedentary; VAS, Visual Analogue Scale; VIC, Victoria; WC, waist circumference.

### Latent profile analysis

A comparison of fit indicators for the VVV models with one to four profiles is presented in online supplementary file 2, table S4). The VVV models consistently had better fit indicators compared with the EEE (online supplementary file 2, figure S1 for comparisons), EEI and VVI models (not presented). The VVV solutions with more than four profiles resulted in profiles representing less than 5% of the sample (n<30) and were therefore not considered.^36^ Based on the fit indicators of the remaining one- to four-profile solutions, the three- and four-profile solutions were deemed better fits compared with the one- and two-profile solutions. Authors (AM, AMCA, DD, JS, KDH, NDR, SJJMV; 21 August 2024) unanimously decided that the four-profile solution was the best option based on model fit indices and interpretability. Classifications derived from this solution were compared with those from the four-profile solution using log-transformed mean bout durations. There was high agreement between profile assignment (Kappa: 0.76^38^). Since the log-transformed bout duration did not yield better model probabilities, the original four-profile model was adopted due to easier interpretability.

**Figure 1:**
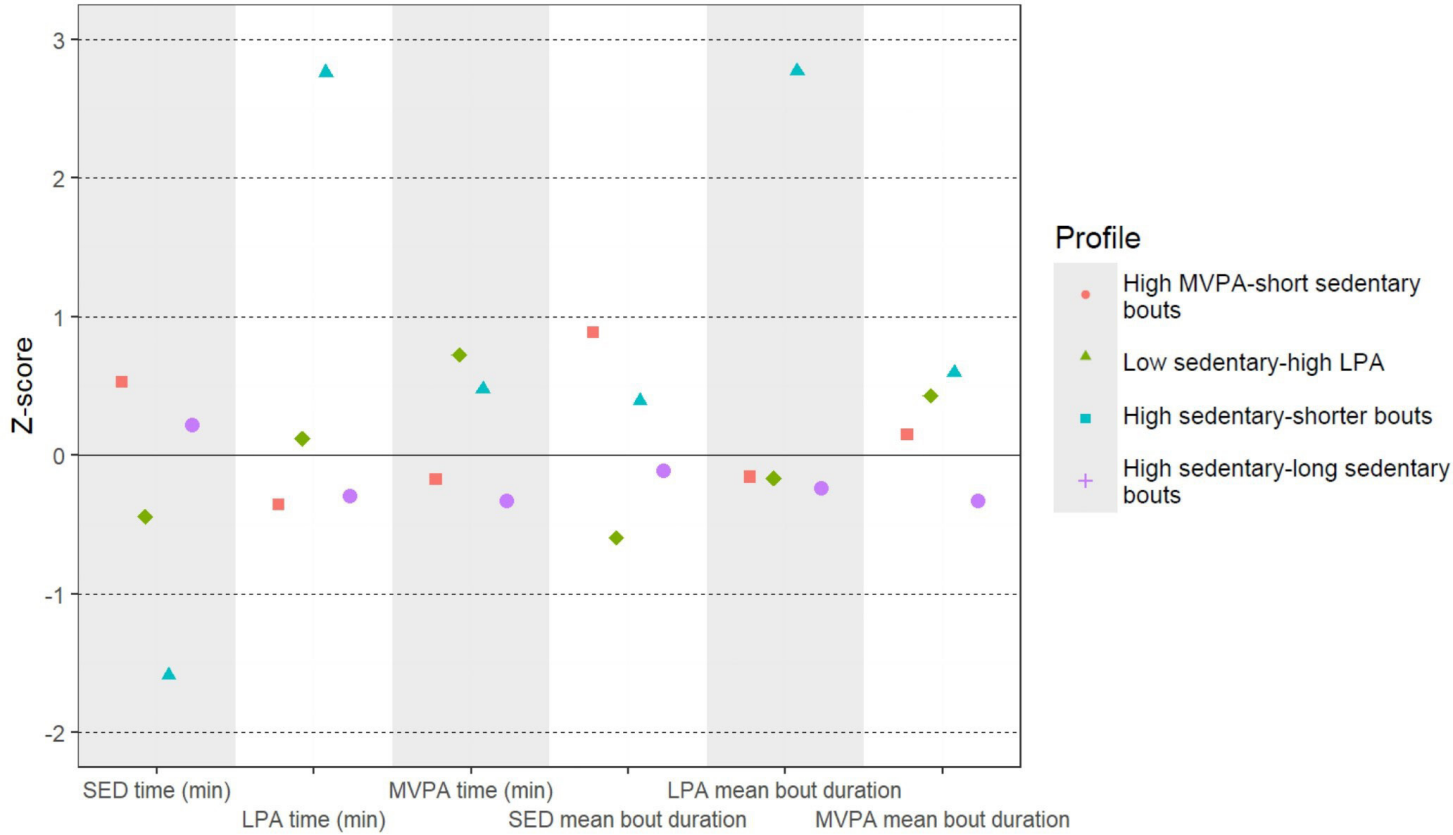
Z-scores of total time and mean bout durations of SED, LPA, and MVPA at baseline among the four distinct profiles Z-scores were calculated by centring values around the mean and dividing by the sample standard deviation, using arithmetic means instead of compositional means for the purpose of creating the figure. LPA, light-intensity physical activity; MVPA, moderate- to vigorous-intensity physical activity; SED, sedentary.

The four unique profile characteristics are displayed (figure 1 and online supplementary table S5) in online supplemental file 2. Specifically, the *high MVPA-short sedentary bout* profile (n=184) exhibited the highest MVPA but also had high sedentary time comparable to the *high sedentary-shorter bouts* profile and the *high sedentary-long sedentary bout* profile, although accrued in shorter durations on average. This profile was comprised predominantly of boys (62%). The *low sedentary-high LPA profile* (n=54) had notably lower sedentary time, as well as more accumulated LPA and longer mean LPA bouts compared with all other profiles. This profile included the youngest participants with the highest proportion of low SES and inner regional area participants, yet were almost exclusively from NSW. The *high sedentary-shorter bouts* (n=405) and the *high sedentary-long sedentary bout* profiles (n=149) had comparably high amounts of sedentary time; however, the *high sedentary-shorter bouts* profile had shorter mean sedentary bouts compared with the *high sedentary-long sedentary bout* profile. Whereas the *high sedentary-shorter bouts* profile consisted predominantly of girls, the *high sedentary-long sedentary bout* profile included mostly boys (66%). Additional classification plots visualising how the four profiles are distributed across the latent profile input variables are provided in online supplementary file 2, figure S2.

### Associations between pattern profiles and health outcomes at baseline and 12-month follow-up

Table 2 and table 3 show the baseline and 12-month associations between the derived latent profiles and health outcomes. The *high sedentary-long sedentary bout* profile was selected as the reference profile, as they were considered the least favourable movement profile (online supplementary file 2, table S5). Compared with the *high sedentary-long sedentary bout* profile, the *high MVPA-short sedentary bout* profile had lower zBMI and zWC, indicating more favourable adiposity levels. However, the omnibus Wald test for differences across profiles indicated uncertainty in the overall profile effect (χ²(3)=4.68, p=0.20), zWC (χ²(3)=7.02, p=0.07). No further cross-sectional associations between the profiles and health outcomes were observed. In addition, no associations between the profiles and health outcomes were observed in the 12-month follow-up models; however, the *high sedentary-shorter bouts* profile had a lower reported HRQoL although the CIs included zero (p=0.06; 95% CI −0.05 to 0.00). Wald test for longitudinal associations showed no evidence of a longitudinal profile effect across outcomes. Sensitivity analysis excluding children identified as underweight based on their zBMI scores or those in the intervention schools did not change the results.

**Table 2:** Cross-sectional associations between latent profiles and health outcomes

	zBMI (n=770)		zWC (n=776)		HRQoL score (n=767)		Perceived health (n=772)	
**Profile**	**β (CI**)	**P value**	**β (CI**)	**P value**	**β (CI**)	**P value**	**β (CI**)	**P value**
High sedentary-long sedentary bout	Reference		Reference		Reference		Reference	
High MVPA-short sedentary bout	***−*0.27 (*−*0.51 to −0.02**)	**0.03**	***−*0.28 (*−*0.51 to −0.05**)	**0.02**	0.007 (*−*0.02 to 0.04)	0.62	0.40 (*−*3.59 to 4.46)	0.83
Low sedentary-high LPA	*−*0.15 (*−*0.50 to 0.20)	0.40	*−*0.05 (*−*0.37 to 0.28)	0.75	0.0001 (*−*0.04 to 0.04)	0.99	*−*2.75 (*−*8.51 to 2.92)	0.39
High sedentary-shorter bouts	*−*0.13 (*−*0.34 to 0.08)	0.22	*−*0.07 (*−*0.27 to 0.13)	0.50	*−*0.006 (*−*0.03 to 0.02)	0.64	*−*1.53 (*−*4.99 to 2.01)	0.46
Omnibus Wald tests	χ²(3)=4.68	*0.20*	*χ²(3)=7.02*	*0.07*	*χ²(3)=1.25*	*0.74*	*χ²(3)=1.84*	*0.61*

Bold values indicate estimates for which the 95% CI does not include zero (p<0.05).

Linear mixed effect regression models were run between the profiles as the categorical exposure variable and continuous adiposity, composite HRQoL score and perceived health at baseline. Models were adjusted for gender, age (only for HRQoL score and perceived health VAS scale), socioeconomic status, and accounted for nesting in schools.The *high sedentary-long sedentary bout *profile was selected as the reference profile as they were considered the profile with the least favourable total times and mean bout durations of sedentary time, LPA and MVPA.

HRQoL, health-related quality of life; LPA, light-intensity physical activity; MVPA, moderate- to vigorous-intensity physical activity; VAS, Visual Analogue Scale; zBMI, age- and sex-standardised body mass index; zWC, age- and sex-standardised waist circumference.

**Table 3:** 12-month follow-up associations between latent profiles and health outcomes

	zBMI (n=437)		zWC (n=440)		HRQoL score (n=548)		Perceived health (n=555)	
**Profiles**	**β (CI**)	**P value**	**β (CI**)	**P value**	**β (CI**)	**P value**	**β (CI**)	**P value**
High sedentary-long sedentary bout	Reference		Reference		Reference		Reference	
High MVPA-short sedentary bout	*−*0.04 (*−*0.15 to 0.08)	0.50	*−*0.04 (*−*0.22 to 0.14)	0.66	*−*0.004 (*−*0.03 to 0.02)	0.77	2.08 (*−*2.12 to 6.28)	0.34
Low sedentary-high LPA	0.05 (*−*0.10 to 0.21)	0.52	*−*0.02 (*−*0.27 to 0.24)	0.89	0.004 (*−*0.03 to 0.04)	0.84	0.08 (*−*6.10 to 6.26)	0.98
High sedentary-shorter bouts	*−*0.001 (*−*0.09 to 0.10)	0.99	*−*0.02 (*−*0.17 to 0.14)	0.84	*−*0.022 (*−*0.05 to 0.00)	0.06	*−*0.04 (*−*3.73 to 3.66)	0.98
Omnibus Wald tests	*χ²(3)=1.37*	0.67	*χ²(3)=0.21*	0.98	*χ²(3)=5.79*	0.12	*χ²(3)=1.62*	0.66

Linear mixed-effect regression models were run between the profiles as the categorical exposure variable and continuous adiposity, composite HRQoL score and perceived health at baseline. Models were adjusted for gender and age (only for HRQoL score and perceived health VAS scale), socioeconomic status, baseline health outcomes, intervention arm and accounted for nesting in schools.The *high sedentary-long sedentary bout *profile was selected as the reference profile, as they were considered the profile with the least favourable total times and mean bout durations of sedentary time, LPA and MVPA.

HRQoL, health-related quality of life; LPA, light-intensity physical activity ; MVPA, moderate- to vigorous-intensity physical activity ; SES, socioeconomic status; VAS, Visual Analogue Scale; zBMI, age- and sex- standardised body mass index; zWC, age- and sex-standardised waist circumference.

## Discussion

This study identified four distinct waking movement behaviour profiles among children aged 7–11 years, characterised by varying patterns of sedentary behaviour, LPA and MVPA time across their waking day. The four derived profiles—*high sedentary-long sedentary bout*, *high sedentary-shorter bouts, high MVPA-short sedentary bout and the low sedentary-high LPA*—differed in both the proportions of movement behaviours and their corresponding mean bout durations, as well as differing in child characteristics and their health outcomes. There was limited evidence for overall differences in health outcomes across profiles, with little evidence from pairwise comparisons. Only the *high MVPA-short sedentary bout* profile showed more favourable adiposity levels cross-sectionally (−0.27 zBMI units; 95% CI −0.51 to −0.02) compared with the *high sedentary-long sedentary bout* profile. Although this estimate should be interpreted in the context of limited evidence for overall differences across profiles, the observed difference of approximately 0.27 zBMI units represents around one quarter of an SD and may be clinically meaningful, given evidence that each 1 SD increase in childhood BMI z-score is associated with an approximately 30% higher risk of cancer in adulthood.^39^ At the 12-month follow-up, only the *high sedentary–shorter bouts* profile showed lower reported HRQoL compared with the reference *high sedentary–long sedentary bout* group; however, the CI included the null (95% CI −0.05 to 0.00; p=0.06) and the omnibus test did not suggest overall profile effects, and this finding should therefore be interpreted with caution.

Both the *high MVPA-short sedentary bout* profile and the *low sedentary-high LPA profile* exhibited high MVPA levels compared with the other two groups, almost complying with current physical activity guidelines recommending at least 60 min of MVPA per day for children (achieving on average 59.6 and 56.2 min/d, on average, with 44% and 30% of participants meeting the guidelines, respectively).^6^ The pairwise finding (despite the lack of evidence from overall group omnibus test) that the *high MVPA-short sedentary bout* profile had favourable adiposity markers cross-sectionally than the *high sedentary-long sedentary bout* profile is consistent with previous research showing that MVPA is most strongly and positively associated with improved health outcomes, compared with lower-intensity activities.^2 40^ However, the same did not hold for the *low sedentary-high LPA profile*, who engaged in nearly as high levels of MVPA, yet their health outcomes did not differ from those of the *high sedentary–long sedentary bout* profile. One possible explanation is that replacing sedentary time with LPA may confer relatively smaller benefits for adiposity, as seen in a previous meta-analysis in youth.^40^ Nevertheless, longer-term longitudinal studies are needed to better understand the potential health implications of different movement behaviour profiles.

The *high sedentary-long sedentary bout* and the *high sedentary-shorter bouts* profiles were similar in their high levels of daily sedentary behaviour and low levels of physical activity across intensities; however, exploratory pairwise comparisons showed that only the *high sedentary-shorter bouts* profile reported lower HRQoL compared with the reference *high sedentary-long sedentary bout* group. It has to be noted that this profile comprised approximately half of the sample, suggesting that observed associations may reflect characteristics of the typical children in this age group^41^ rather than effects attributable solely to their movement patterns. However, it may suggest that the way sedentary behaviour is accumulated, specifically, the mean duration of sedentary bouts, may have differential effects on health. To further investigate this, it is necessary to employ study designs that experimentally manipulate bout lengths so that accurate conclusions about the health impacts of such patterns can be made. Another potential explanation for these unexpected findings could be gender differences, as the *high sedentary-long sedentary bout* profile contained more girls than the *high sedentary-shorter bouts* profile; however, gender was not formally tested as an effect modifier alongside the group comparisons presented in the present analyses, and this interpretation should therefore be considered exploratory. Previous evidence that girls in this age group experience more self-reported problems and less pain compared with boys, which are individual domains of HRQoL.^42^ In contrast, SES was broadly comparable across profiles (ranging between 48% and 63%), suggesting that SES is unlikely to account for the observed differences in health outcomes, despite its well-established association with physical activity.^43^

### Research and policy implications

The findings from this study underscore the importance of recognising the diversity in children’s movement behaviour patterns when developing health interventions and policies. The identification of distinct profiles impacts how interventions should be tailored to address these specific movement behaviours and consider their activity patterns, rather than only aiming to increase overall activity levels. An example of how this could be done is by providing various opportunities for children to be active throughout the day, such as through whole-school movement initiatives, encouraging children to reach sufficient MVPA throughout the day with limited prolonged sitting—aiming for a replication of the *high MVPA-short sedentary bout* profile’s behaviour which was associated with best adiposity outcomes, although cross-sectionally. Specific promising school-based initiatives include incorporating regular classroom activity breaks to interrupt prolonged sitting^44^ and providing equipment during recess to increase engagement in MVPA.^45^ These initiatives should be further co-designed with children, parents, and teachers to ensure feasibility, acceptability and sustained uptake.

### Limitations

A number of limitations should be considered when interpreting these findings. First, this study did not account for sleep behaviours and diet, which are known to influence both physical activity patterns and health outcomes long-term in children.^6^ Second, only 57.8% of participants with parental consent provided valid accelerometry data at baseline, and were therefore included in the latent profile analysis. While the participants who provided data at baseline did not differ largely from those in the longitudinal analytical sample, they were less likely to be from a major city, younger and have a lower baseline zWC, which may have led to some bias in the results. Third, the *low sedentary-high LPA profile* was relatively small compared with the other profiles and included mostly participants from the control group, which may limit the generalisability of this profile. The downstream analyses were also based on most-likely class assignment and did not account for uncertainty in profile membership, which may have biased estimates toward statistical significance and should be considered when interpreting these findings. Fourth, it is important to note that COVID-19 impacted the TransformUs trial significantly. Anthropometric data were not collected for a subset of participants at follow-up due to COVID-19-related disruptions, resulting in missing adiposity outcomes for a subset of participants. This reduced the sample size for longitudinal adiposity analyses and may have limited statistical power and representativeness, with the potential for selection bias if children with missing follow-up measurements differed systematically from those with complete data.^46^ Moreover, the study’s HRQoL measures were taken during the COVID-19 pandemic, and it is possible that this influenced children’s responses, particularly if the timing of data collection coincided with periods of lockdown or school closures, which could have impacted both physical activity patterns and perceptions of health and well-being. Fifth, it has to be noted that in ‘otherwise healthy’ general young populations, there is a strong ceiling effect for the 'no problems’ level in mobility, independence, and daily living activities. The EQ-5D-Y may lack sensitivity in generally healthy child populations and therefore may have had limited ability to detect associations in the current study. Sixth, future studies could consider applying Rasch modelling to HRQoL measures to better account for ceiling effects. Lastly, as latent profile memberships were based on most likely class assignment, classification uncertainty was not accounted for into subsequent models.

## Conclusion

Four distinct movement profiles were identified. Few pairwise differences between health outcomes were observed. While MVPA remains a key factor for promoting healthy body weight, our findings suggest that a variety of movement patterns—including those characterised by lower sedentary time and higher LPA—may support health in children. Further research including 24-hour wear protocols, to also incorporate sleep as an important part of the day, is needed to refine these recommendations and explore the long-term effects of different movement patterns on children’s health.

## Supplementary material

10.1136/bmjopen-2025-109130 online supplemental file 1

10.1136/bmjopen-2025-109130 online supplemental file 2

## Data Availability

Data sharing not applicable as no datasets generated and/or analysed for this study. Data are available upon reasonable request.
