# Supplementary material for: Latent profiles of movement behaviour compositions and their associations with adiposity and health-related quality of life in Australian children: a cross-sectional and 12-month longitudinal study
Source: BMJ Open. 2026 Jun 8;16(6):e109130. doi: 10.1136/bmjopen-2025-109130 (PMC13250231; doi:10.1136/bmjopen-2025-109130)
Supplement: online supplemental file 2 [file bmjopen-16-6-s002.pdf]

Table S1. STROBE Statement

	Item No.	Recommendation	Page No.	Relevant text from manuscript
**Title and abstract**	1	(*a*) Indicate the study's design with a commonly used term in the title or the abstract	1	Latent profiles of movement behaviour compositions and their associations with adiposity and health-related quality of life in Australian children: A cross-sectional and 12-month longitudinal study
		(*b*) Provide in the abstract an informative and balanced summary of what was done and what was found	3	Objectives: To identify … registered with the Australian Clinical Trials Registry (ACTRN12617000204347; 1 April 2017).
**Introduction**				
Background/rationale	2	Explain the scientific background and rationale for the investigation being reported	5-6	The time children … mean bout durations.
Objectives	3	State specific objectives, including any prespecified hypotheses	6	The aim of … markers and HRQoL.
**Methods**				
Study design	4	Present key elements of study design early in the paper	6	Analyses were conducted … n=582 in NSW]).
Setting	5	Describe the setting, locations, and relevant dates, including periods of recruitment, exposure, follow-up, and data collection	6-7	Analyses were conducted … through parent survey.
Participants	6	(*a*) *Cohort study*—Give the eligibility criteria, and the sources and methods of selection of participants. Describe methods of follow-up<br>*Case-control study*—Give the eligibility criteria, and the sources and methods of case ascertainment and control selection. Give the rationale for the choice of cases and controls	6-7	For the effectiveness … through parent survey.

		Cross-sectional study—Give the eligibility criteria, and the sources and methods of selection of participants		
		(*b*) *Cohort study*—For matched studies, give matching criteria and number of exposed and unexposed<br>*Case-control study*—For matched studies, give matching criteria and the number of controls per case	N/A	N/A
Variables	7	Clearly define all outcomes, exposures, predictors, potential confounders, and effect modifiers. Give diagnostic criteria, if applicable	6-8	Movement behaviour (via … remote/very remote areas.
Data sources/ measurement	8*	For each variable of interest, give sources of data and details of methods of assessment (measurement). Describe comparability of assessment methods if there is more than one group	6-8	Movement behaviour (via … remote/very remote areas.
Bias	9	Describe any efforts to address potential sources of bias	7<br><br>10	Of the 1,370 … Supplementary File 2, Table S2.<br><br>Baseline models were … outcome at follow-up).
Study size	10	Explain how the study size was arrived at	7<br><br>8<br><br>Table 1	Of the 1,370 … Supplementary File 2, Table S2.<br><br>Data from all … the latent profile analysis.<br><br>Table 1
Quantitative variables	11	Explain how quantitative variables were handled in the analyses. If applicable, describe which groupings were chosen and why	6-8	Movement behaviour (via … remote/very remote areas.
Statistical methods	12	(*a*) Describe all statistical methods, including those used to control for confounding	8-10	Statistical analyses were … in diagnostic plots.
		(*b*) Describe any methods used to examine subgroups and interactions	N/A	N/A
		(*c*) Explain how missing data were addressed	7	This led to … power and representativeness.
		(*d*) *Cohort study*—If applicable, explain how loss to follow-up was addressed<br>*Case-control study*—If applicable, explain how matching of cases and controls was addressed	10	Linear mixed-effect regression …the school level.

		Cross-sectional study—If applicable, describe analytical methods taking account of sampling strategy		
		(*e*) Describe any sensitivity analyses	10	Sensitivity analysis were … schools at follow-up.
**Results**				
Participants	13*	(a) Report numbers of individuals at each stage of study—eg numbers potentially eligible, examined for eligibility, confirmed eligible, included in the study, completing follow-up, and analysed	11	Participant characteristics are … 46.2 minutes/day (SD = 18.5).
		(b) Give reasons for non-participation at each stage	19-20<br>Table 1	Anthropometric data were … with complete data (46).
		(c) Consider use of a flow diagram	N/A	N/A
Descriptive data	14*	(a) Give characteristics of study participants (eg demographic, clinical, social) and information on exposures and potential confounders	11<br><br>Table 1	Participant characteristics are … 46.2 minutes/day (SD = 18.5).<br><br>Table 1
		(b) Indicate number of participants with missing data for each variable of interest	Table 1	Table 1
		(c) *Cohort study*—Summarise follow-up time (eg, average and total amount)	6-8	Movement behaviour (via … remote/very remote areas.
Outcome data	15*	*Cohort study*—Report numbers of outcome events or summary measures over time	6-8	Movement behaviour (via … remote/very remote areas.
		Case-control study—Report numbers in each exposure category, or summary measures of exposure	N/A	N/A
		Cross-sectional study—Report numbers of outcome events or summary measures	N/A	N/A
Main results	16	(*a*) Give unadjusted estimates and, if applicable, confounder-adjusted estimates and their precision (eg, 95% confidence interval). Make clear which confounders were adjusted for and why they were included	Table 2, Table 3	Confounder-adjusted estimates are presented in Table 2 and Table 3 (including footnotes for adjustments).
		(*b*) Report category boundaries when continuous variables were categorized	N/A	N/A
		(*c*) If relevant, consider translating estimates of relative risk into absolute risk for a meaningful time period	N/A	N/A

Continued on next page

Other analyses	17	Report other analyses done—eg analyses of subgroups and interactions, and sensitivity analyses	15	Sensitivity analysis excluding children … change the results.
**Discussion**				
Key results	18	Summarise key results with reference to study objectives	18	This study identified … interpreted with caution.
Limitations	19	Discuss limitations of the study, taking into account sources of potential bias or imprecision. Discuss both direction and magnitude of any potential bias	19-20	A number of … into subsequent models.
Interpretation	20	Give a cautious overall interpretation of results considering objectives, limitations, multiplicity of analyses, results from similar studies, and other relevant evidence	18-20	This study identified … into subsequent models.
Generalisability	21	Discuss the generalisability (external validity) of the study results	20	The findings from … into subsequent models.
**Other information**				
Funding	22	Give the source of funding and the role of the funders for the present study and, if applicable, for the original study on which the present article is based	4	The TransformUs trial … of this paper.

*Give information separately for cases and controls in case-control studies and, if applicable, for exposed and unexposed groups in cohort and cross-sectional studies.

Note: An Explanation and Elaboration article discusses each checklist item and gives methodological background and published examples of transparent reporting. The STROBE checklist is best used in conjunction with this article (freely available on the Web sites of PLoS Medicine at http://www.plosmedicine.org/, Annals of Internal Medicine at http://www.annals.org/, and Epidemiology at http://www.epidem.com/). Information on the STROBE Initiative is available at www.strobe-statement.org.

Table S2. Description of accelerometer data processing

Data were excluded from analyses if post-calibration error was greater than 0.02 g or fewer than 8 hours of wear-time were recorded by the monitor during a waking day(1). Non-wear was estimated based on statistics derived from a rolling time window. It was defined as either when, for at least two out of the three accelerometer axes, the standard deviation was less than 13 milli gravity-based acceleration units (m*g*) or if the value range of accelerations for each axis was less than 50 m*g*(2). Since participants did not wear the monitors overnight, the “not worn” protocol was selected in GGIR to exclude the sustained inactivity bouts with close to zero movement. Previously validated acceleration intensity thresholds were used to distinguish between time spent sedentary (<32.6 m*g*(3)), light-intensity physical activity (LPA; 32.6-<142.6 m*g*(4, 5)), moderate-intensity (142.6-<464.6 m*g*(4, 5)) and vigorous-intensity physical activity (>464.6 m*g*(4, 5)). Total daily MVPA was calculated as the sum of MPA and VPA. The mean durations of sedentary time, LPA, and MVPA were calculated for each day. Only data from participants with at least four valid wear days (inclusive of at least one weekend day) were included in the analytic sample(6).Weighted averages across the wear days weighting weekdays and weekend days 5:2 for all variables (durations and bouts) were then calculated.

References

1. Cain KL, Sallis JF, Conway TL, Van Dyck D, Calhoon L. Using accelerometers in youth physical activity studies: A review of methods. J Phys Act Health. 2013;10(3):437-50.
2. van Hees VT, Gorzelniak L, Dean Leon EC, Eder M, Pias M, Taherian S, et al. Separating movement and gravity components in an acceleration signal and implications for the assessment of human daily physical activity. PLoS One. 2013;8(4):e61691.
3. Hurter L, Fairclough SJ, Knowles ZR, Porcellato LA, Cooper-Ryan AM, Boddy LM. Establishing Raw Acceleration Thresholds to Classify Sedentary and Stationary Behaviour in Children. Children (Basel). 2018;5(12).
4. Hildebrand M, Hansen BH, van Hees VT, U E. Evaluation of raw acceleration sedentary thresholds in children and adults. Scand J Med Sci Sports. 2016;27(12):1814-23.
5. Hildebrand M, van Hees VT, Hansen BH, Ekelund U. Age group comparability of raw accelerometer output from wrist- and hip-worn monitors. Med Sci Sports Exerc. 2014;46:1816-24.
6. Mattocks C, Ness AR, Leary SD, Tilling K, Blair SN, Shield J, et al. Use of accelerometers in a large field-based study of children: Protocols, design issues, and effects on precision. J Phys Act Health. 2008;5 (Supplement 1):S98-S111.

Table S3. CHAMP: CHecklist for statistical Assessment of Medical Papers

Design and conduct				
1. Clear description of the goal of research, study objective(s), study design, and study population	Yes	Unclear	No
2. Clear description of outcomes, exposures/treatments and covariates, and their measurement methods	Yes	Unclear	No
3. Validity of study design	Yes	Unclear	No
4. Clear statement and justification of sample size	Yes	Unclear	No
5. Clear declaration of design violations and acceptability of the design violations	Yes	N/A	No
6. Consistency between the paper and its previously published protocol	Yes	Unclear	No
Data analysis			
7. Correct and complete description of statistical methods	Yes	Unclear	No
8. Valid statistical methods used and assumptions outlined	Yes	Unclear	No
9. Appropriate assessment of treatment effect or interaction between treatment and another covariate	Yes	Unclear	No
10. Correct use of correlation and associational statistical testing	Yes	Unclear	No
11. Appropriate handling of continuous predictors	Yes	Unclear	No
12. Confidence intervals do not include impossible values	Yes	Unclear	No
13. Appropriate comparison of baseline characteristics between the study arms in randomized trials	Yes	Unclear	No
14. Correct assessment and adjustment of confounding	Yes	Unclear	No
15. Avoiding model extrapolation not supported by data	Yes	Unclear	No
16. Adequate handling of missing data	Yes	Unclear	No
Reporting and presentation			
17. Adequate and correct description of the data	Yes	Unclear	No
18. Descriptive results provided as occurrence measures with confidence intervals, and analytic results provided as association measures and confidence intervals along with P-values	Yes	Unclear	No
19. Confidence intervals provided for the contrast between groups rather than for each group	Yes	Unclear	No
20. Avoiding selective reporting of analyses and P-hacking	Yes	Unclear	No
21. Appropriate and consistent numerical precisions for effect sizes, test statistics, and P-values, and reporting the P-values rather their range	Yes	Unclear	No
22. Providing sufficient numerical results that could be included in a subsequent meta-analysis	Yes	Unclear	No
23. Acceptable presentation of the figures and tables	Yes	Unclear	No
Interpretation			
24. Interpreting the results based on association measures and 95% confidence intervals along with P-values, and correctly interpreting large P-values as indecisive results, not evidence of absence of an effect	Yes	Unclear	No
25. Using confidence intervals rather than post-hoc power analysis for interpreting the results of studies	Yes	Unclear	No
26. Correctly interpreting occurrence or association measures	Yes	Unclear	No
27. Distinguishing causation from association and correlation	Yes	Unclear	No
28. Results of pre-specified analyses are distinguished from the results of exploratory analyses in the interpretation	Yes	Unclear	No
29. Appropriate discussion of the study methodological limitations	Yes	Unclear	No
30. Drawing only conclusions supported by the statistical analysis and no generalization of the results to subjects outside the target population	Yes	Unclear	No

Table S4. Comparison of fit indicators for VVV solutions with one to four profiles

Number of profiles	BIC	AIC	SABIC	CAIC	AWE	ICL	Entropy	LL	BLRTS (p-value)	Observations per profile
1	-8790	8704	8733	8817	8991	-8797	0	-4332	NA	792
2	-3791	3599	3661	3832	4188	-3809	**0.96**	-1759	5146 (0.001)	679/113
3	-3260	2970	3063	3322	**3860**	**-3333**	0.89	-1423	671 (0.001)	598/59/135
4	**-3163**	**2775**	**2899**	**3246**	3966	-3389	0.78	**-1304**	**238 (0.001)**	184/54/405/149

Bolded values denote the value corresponding to the “best” model according to each fit indicator.

AIC: Akaike’s information criteria; AWE: Adjusted Weighted Evidence; BIC: Bayesian information criteria; BLRTS: Bootstrapped Likelihood Ratio Test Statistics; CAIC: Consistent Akaike Information Criterion; ICL: Integrated Completed Likelihood; LL: Loglikelihood; SABIC: Sawa’s Adjusted Bayesian Information Criterion.

Note: Only VVV models (varying volume, varying shape, varying orientation) are presented in this table; these models consistently had the best fit compared to the EEE (Supplementary File 2, Figure S1 for comparisons) and EEI and VVI models (not presented)

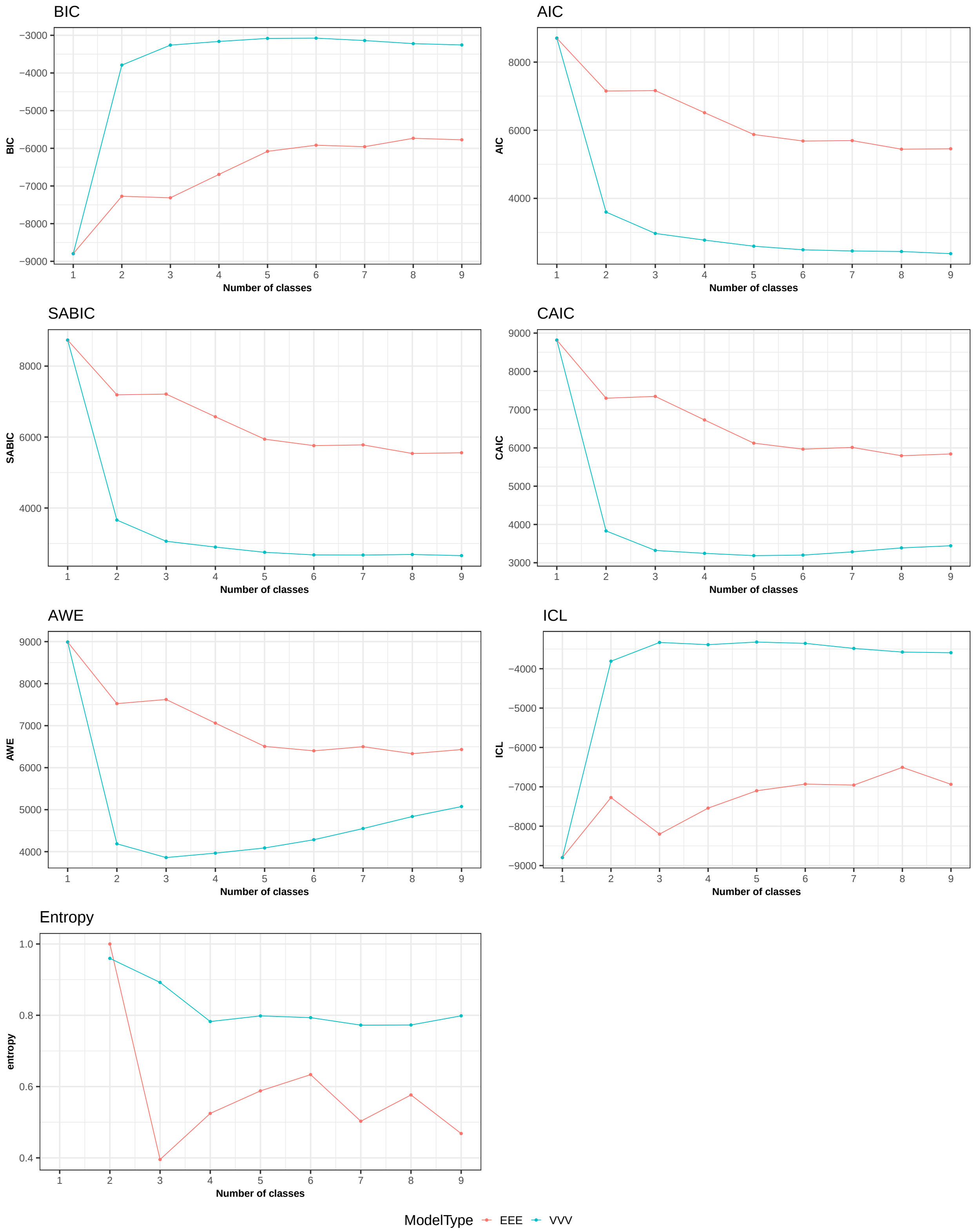

Figure S1. Comparison of fit indicators for EEE and VVV models with one to nine profiles

AIC: Akaike's information criteria; AWE: Adjusted Weighted Evidence; BIC: Bayesian information criteria; BLRTS: Bootstrapped Likelihood Ratio Test Statistics; CAIC: Consistent Akaike Information Criterion; ICL: Integrated Completed Likelihood; LL: Loglikelihood; SABIC: Sawa's Adjusted Bayesian Information Criterion.

Table S5. Participant baseline characteristics for the latent profiles

Profile	n	SED time (min)[A]	LPA time (min)[A]	MVPA time (min)[A]	Mean duration SED bouts (min)	Mean duration LPA bouts (min)	Mean duration MVPA bout (min)	Age (yrs)	Gender (% girls)	SES (% high)	Geographical location (% major city)	Intervention arm (% VIC)	% Meeting the MVPA guidelines [6]
**High sedentary-long sedentary bout**	149	613.4	110.3	36.2	20.6 (5.7)	2.4 (0.3)	2.5 (0.6)	**9.2**	**44%**	59%	**75%**	**64%**	20.1
**High MVPA-short sedentary bout**	184	543.9	156.4	59.6	11.5 (1.8)	2.4 (0.2)	2.6 (0.4)	**9.0**	**38%**	63%	**73%**	**70%**	44.0
**Low sedentary-high LPA**	54	355.9	347.9	56.2	17.6 (16.5)	9.1 (5.8)	2.7 (0.8)	**8.8**	**56%**	48%	**59%**	**4%**	29.6
**High sedentary-shorter bouts**	405	601.9	120.1	37.9	14.5 (2.4)	2.3 (0.2)	2.3 (0.3)	**9.1**	**59%**	56%	**77%**	**53%**	6.42

Bolded values indicate differences between profiles for which the 95% confidence interval does not include zero ($p < 0.05$). Test statistics for comparison between profiles: Gender - X-squared =27.1, $p<0.001$; SES - X-squared=4.6, $p=0.2038$; Geography - X-squared=8.4, $p=0.038$; Age - F value=5.6, $p<0.001$; Intervention arm - X-squared=46.0, $p<0.001$. Tests were not conducted for proportion of participants meeting the guidelines.

[A] Values represent compositional means for SED, LPA and MVPA, closed to 760min.

[B] Values represent arithmetic means.

LPA: Light-intensity physical activity; MVPA: Moderate- to vigorous-intensity physical activity; SED: Sedentary; SES: Socioeconomic status; VIC: Victoria.

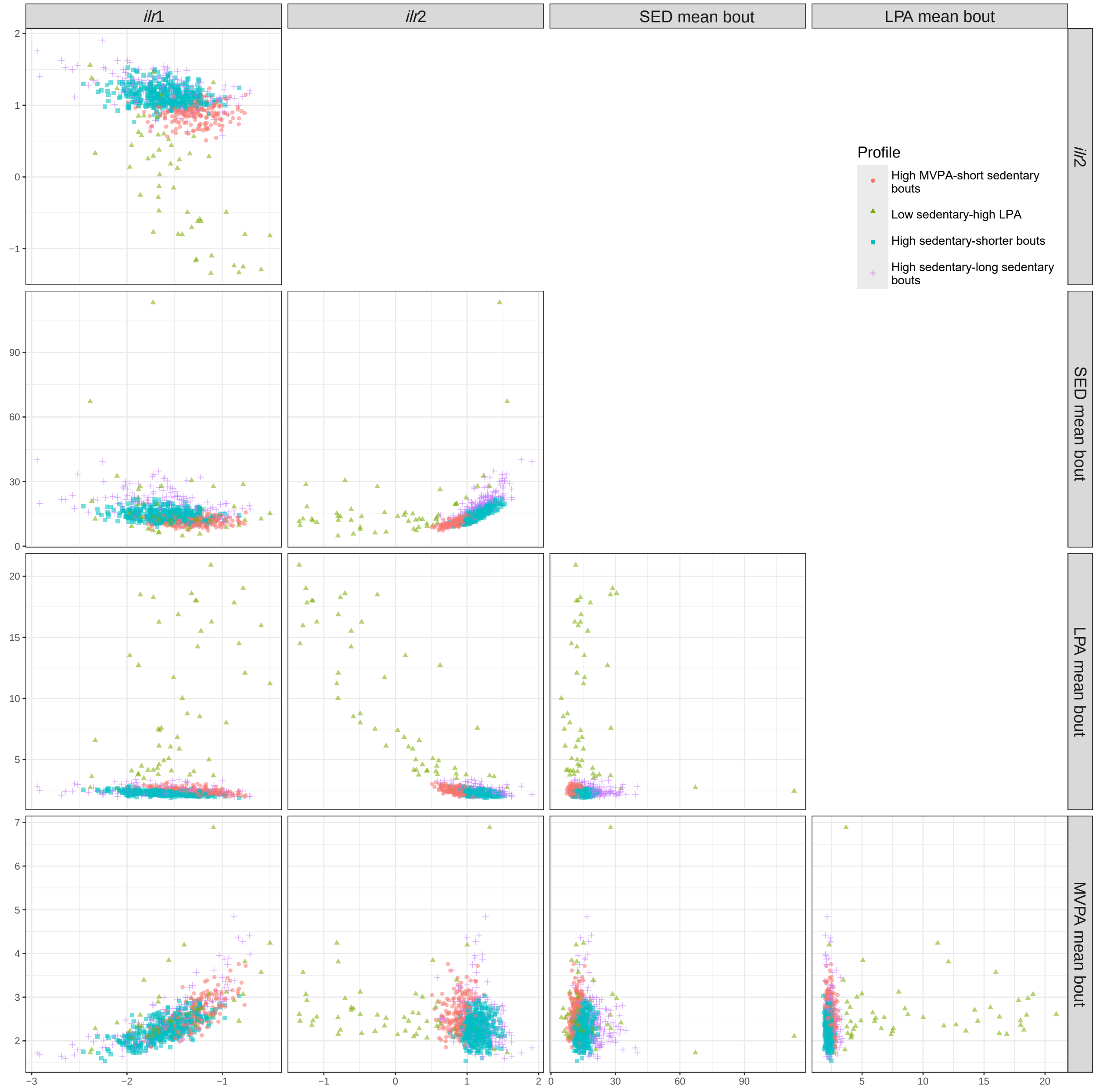

Figure S2. Classification plots

*ilr*s: Isometric Log Ratios; LPA: Light-intensity Physical Activity; MVPA: moderate- to vigorous-intensity physical activity; SED: Sedentary.
