# Supplementary material for: Latent profiles of movement behaviour compositions and their associations with adiposity and health-related quality of life in Australian children: a cross-sectional and 12-month longitudinal study
Source: BMJ Open. 2026 Jun 8;16(6):e109130. doi: 10.1136/bmjopen-2025-109130 (PMC13250231; doi:10.1136/bmjopen-2025-109130)
Supplement: Supplementary file 3 [file bmjopen-2025-109130.reviewer_comments.pdf]

PEER REVIEW HISTORY

BMJ Open publishes all reviews undertaken for accepted manuscripts. Reviewers are asked to complete a checklist review form and are provided with free text boxes to elaborate on their assessment. These free text comments are reproduced below.

ARTICLE DETAILS

Title (Provisional)

Latent profiles of movement behaviour compositions and their associations with adiposity and health-related quality of life in Australian children: A cross-sectional and 12-month longitudinal study

Authors

Verswijveren, Simone J.J.M.; Miatke, Aaron; Hesketh, Kylie D; Ridgers, Nicola D.; Contardo Ayala, , Ana María; Dumuid, Dorothea; Timperio, Anna; Rasmussen, Charlotte; Stanford, Tyman E; Gusi , N; Salmon, Jo

---

VERSION 1 - REVIEW

**Reviewer**	**1**
**Name**	**Alkazemi, Dalal**
**Affiliation**	**Kuwait University, Food Science and Nutrition**
**Date**	**14-Nov-2025**
**COI**	**None**

The manuscript is difficult to follow in its current form. The narrative is fragmented, key points are repeated, and the flow between sections is uneven. The article would benefit from a clearer structure, reduced redundancy, simplified profile descriptions, and a more streamlined methods section to strengthen readability and interpretation.

Specific comments by line number

Abstract (Lines 54–62)

– The use of animal-based profile labels requires clear justification; as written, these metaphors undermine the scientific tone.

– Key outcomes (BMI, WC, HRQoL) are not reported and should be included.

– The conclusion does not reflect the presented findings and should be revised accordingly.

Summary Box (Lines 71–90)

– The bullets should more directly reflect the study question and added value.

– Methodological concepts (e.g., variable-centered approaches, population-distribution assumptions) are introduced without grounding and appear disconnected.

– The reference to population distribution is unnecessary in this context.

Introduction

– The rationale for “distinct profiles of compositional movements” is unclear; explain why these profiles represent meaningful archetypes.

– Lines 94–115 cover an age range of 5–17 years, which is inconsistent with the study focus of 7–11 years; clarify this early.

– Lines 104–112 repeat similar points on sedentary behavior; merge these statements.

– Lines 119–123 and 124–129 duplicate explanations of variable vs. person-centered approaches; combine for clarity.

– Lines 131–135 present the key methodological gap (bout-duration analysis); consider placing this earlier in the introduction.

Methods

– Lines 173–175: Missing anthropometry due to COVID-19 is noted but not explained; clarify its extent and potential impact.

– Lines 178–204: Accelerometer-processing details are excessive for the main text; move most to supplementary material.

– Lines 260–267 combine CoDA transformations, bout metrics, and latent-profile variables; separate these elements to improve readability.

Model Selection and Profiles

– Lines 327–341: Explain the meaning of the covariance structures (VVV vs. EEE) and why VVV was selected.

– Lines 342–354 rely heavily on metaphors; provide quantitative contrasts (e.g., effect sizes, standardized values).

– Table 3 mixes compositional and arithmetic means without labelling; this should be clarified.

Results

– Lines 377–386: The statement of “no further associations” overlooks wide confidence intervals; acknowledge this uncertainty.

Discussion

– Lines 418–424 repeat novelty statements already covered in the introduction; condense to avoid redundancy.

– Lines 433–439 offer speculative explanations for adiposity patterns; revise to present these more cautiously or support with evidence.

– Lines 441–452 attribute differences to gender without analysis; test gender as a modifier or frame this clearly as speculation.

Research and Policy Implications

– Lines 455–465 remain broad; include two concrete examples relevant to school settings.

Limitations

– Lines 469–475 mention possible bias from missing data but do not quantify it; provide a comparison of included vs. excluded participants.

– Lines 480–481 discuss ceiling effects in HRQoL but do not mention analytic alternatives; state whether Rasch modelling was considered.

– Lines 486–489 call for complete 24-hour protocols without linking this to CoDA; clarify why incorporating sleep is essential for 24-hour compositional modelling.

Reviewer **2**

Name **Salway, Ruth**

Affiliation **University of Bristol, Centre for Exercise, Nutrition and Health Sciences**

Date **05-Dec-2025**

COI **None**

This is an interesting and well-written paper that looks at associations between activity profiles and adiposity and quality of life in children. I think this could be a valuable addition to the evidence, but I have a few issues that need to be addressed, mainly around about some of the analysis and the discussion.

1) I don't believe that it is appropriate to include intervention schools at follow-up. Even though this trial was not 'effective' overall, one of the arguments for using latent profile analysis rather than simple PA estimates is that it allows exploration of more complex patterns of activity, and the fact that the programme has previously been found to have an impact on adiposity, sedentary and PA levels makes it extremely likely that it may have impacted these patterns in some way.

2) The bulk of the follow-up data were collected during the COVID pandemic. There has been a lot of evidence that the pandemic affected PA/SB levels and patterns, adiposity and health and well-being, so any 12-month associations are only valid for during the pandemic and cannot be generalised outside of that time. This requires more justification and far more careful interpretation in the discussion, rather than just a brief mention in the limitations.

3) The framing of this study as compositional data analysis is not really justified as it doesn't allow the key feature of CoDA which is to explore the potential impact of reallocation of time from one activity to another. This is simply LPA using combinations of movement behaviours across the day – this is useful but it's not CoDA.

4) The discussion is under-developed and mainly repeats the results, and there is a lack of reference to the wider literature (four references in total, one of which is just to the PA guidelines). This should be expanded. For example: the COVID impact on follow-up as in point 2; the fact that half the children are in the lazing lions profile and so any associations may represent the 'typical' child rather than activity patterns; the role of bouts in the profiles; discussion of gender and SES differences; the fact that hummingbirds are nearly all from one geographical area, suggesting there may be issues around generalisability of the results.

5) The implications suggest that interventions should focus on moving children from one activity profile to another. Firstly this implies a causal direction to the association (and evidence suggests this is bi-directional with a stronger relationship for adiposity leading to lower PA). But secondly, the suggestions – encouraging children to be active throughout the day without prolonged sitting time – are fairly generic and don't really address how you might facilitate this change and how it might differ between bears and lions, for example.
6) There are also several issues around the reporting of the statistical analyses:

   a. More detail is needed in the main text on the latent profile analysis, rather than hidden in the supplementary materials. For example, VVV, EEI etc are referred to in the results but not explained anywhere; how the optimal number of profiles was chosen. What R package was used?
   b. LPA is a probability-based approach. I assume that children have been assigned to the most likely profile for the subsequent analysis; this should be made clear, alongside the limitations of this approach. As the variability is not being accounted for in the subsequent models, this also suggests that caution should be taken in interpreting borderline p-values. Using 'most likely profile' rather than feeding through the variability should also be acknowledged in the limitations.
   c. Line 291 re Tobit model: model choice should based on validity of model assumptions and not on model fit – it doesn't matter how good the fit is of a linear model if it breaks the underlying assumptions because this invalidates any results.
   d. The term 'statistical significance' should be avoided, and instead report estimates and confidence intervals alongside p-values.
   e. There are a lot of p-values reported, and so care should be taken on identifying associations based on borderline p-values. Also analysing the subdomains of the HRQoL feels like fishing for 'significant' associations, especially when there is no evidence of association with HRQoL overall.
   f. In Tables 4 & 5, the profiles are mutually exclusive and so are a categorical variable and so the relevant p-value should be for a Wald test for the category as a whole, and not separate p-values for each profile.

Some minor points:

1) It's not always clear in the introduction which evidence refers to children and young people generally and which relates to the specific age group 7-11 explored in this study.
2) Line 164: Years 3 and 4 (aged 7-11 years) - aren't these ages 8-10?
3) Line 172: presumably measurements were not the same – do you mean measurement protocols (eg days of wear etc) as opposed to data collection protocols?
4) Line 180+ are these wrist or hip worn accelerometers?
5) ln 349 – hummingbirds nearly all from NSW – this does not suggest that these profiles can be interpreted as generalisable across populations then
6) ln 347 – I'm not sure two months younger on average is a difference worth noting.
7) Table 2: please add the fit indicators for more profiles, so it is clear why 4 profiles is to be preferred to more.
8) Line 378: surely the lazing lions as the largest profile (so representing the most typical activity patterns) would make a better reference category? Also 'referent' is the wrong word; should be 'reference'.
9) Tables 4 & 5: it's not clear how to interpret the magnitude of these coefficients – is a difference of 0.27 in zBMI meaningful? Also is perceived health the same as the VAS scale? If so, can you define somewhere/be consistent.
10) Line 430-32: 'almost complying with physical activity guidelines'. This is not true – it's not meaningful to compare a group (profile) average to an individual threshold, and due to variability quite a lot of children in these profiles will achieve 60+mins. It might be useful to actually calculate the proportion of children in each profile who achieve the guidelines.
11) Supplementary Table S2: reference 2 is incomplete, and references for BIC etc are needed. If possible, use references to the original methods rather than to tutorials.
12) No Supplementary Table S3?
13) Supplementary Tables S4 & S5: these would be easier to interpret if they reported odds ratios and confidence intervals (and p-values should be explicitly reported rather than using * for statistical significance).

VERSION 1 - AUTHOR RESPONSE

Reviewer 1 Dr. Dalal Alkazemi, Kuwait University:

1) The manuscript is difficult to follow in its current form. The narrative is fragmented, key points are repeated, and the flow between sections is uneven. The article would benefit from a clearer structure, reduced redundancy, simplified profile descriptions, and a more streamlined methods section to strengthen readability and interpretation.

Thank you for your comments. We have substantially revised the manuscript to improve clarity and flow, profile descriptions, and a more streamlined methods section using the detailed and constructive feedback provided by both reviewers. Some examples of how we have done this:

- *Clarity: We have removed the duplicate information in some of the paragraphs of the introduction, see our responses to comments #12 and #13.*

- *Simplified profile descriptions: We have now removed the animal profile names and instead used names based on their movement behaviours. This has been incorporated throughout the manuscript. E.g.:*

  Four distinct profiles were identified. The high MVPA-short sedentary bout profile (n=184) was characterised by the highest levels of MVPA, moderate sedentary time, and the shortest mean sedentary bout duration. The low sedentary-high LPA profile (n=54) had the lowest sedentary time, the highest LPA, and the longest mean LPA bout duration. Two profiles were characterised by high sedentary time: the high sedentary-long sedentary bout profile (n=149), which had the longest mean sedentary bout durations, and the high sedentary-shorter bouts profile (n=405), which also had high sedentary time but shorter bout durations for all intensities. Whilst the omnibus Wald test for differences across profiles indicated uncertainty in the overall profile effect, the high MVPA-short sedentary bout profile had favourable adiposity levels cross-sectionally compared to the high sedentary-long sedentary bout reference profile in pairwise comparisons. No longitudinal associations were detected. [Page 3, Lines 62-73]

- *Streamlined methods section: In response to reviewer suggestions, we have moved most accelerometer processing details to the Supplementary Materials and separated the statistical methods into multiple clearly defined subsections, each with its own heading, to improve readability. We believe these changes have substantially enhanced the clarity, readability, and interpretability of the manuscript.*

Overall, we believe these changes have substantially improved the readability and interpretability of the manuscript. All other comments have been specifically addressed below.

<u>Abstract:</u>

2) Abstract (Lines 54–62): The use of animal-based profile labels requires clear justification; as written, these metaphors undermine the scientific tone.

We have removed the animal-based profiles and have used clearer profile names throughout the manuscript. An example can be found in comment #3.

3) Abstract (Lines 54–62): Key outcomes (BMI, WC, HRQoL) are not reported and should be included.

We have now included these in the abstract as follows:

Primary outcomes were age- and sex-standardized body mass index, waist circumference, and parent-reported health-related quality of life at baseline. Secondary outcomes were the same measures assessed at 12-month follow-up. [Page 3, Lines 58-61]

4) Abstract (Lines 54–62): The conclusion does not reflect the presented findings and should be revised accordingly.

We have rewritten the conclusion to reflect our findings better as follows:

Conclusions: Four distinct movement profiles were identified, each with unique implications for health. While MVPA remains a key factor for promoting healthy body weight, our findings suggest that a variety of movement patterns - including those characterized by lower sedentary time and higher LPA - may also support health in children. [Page 3, Lines 74-77]

Summary box:

5) Summary Box (Lines 71–90): The bullets should more directly reflect the study question and added value.

In line with the Editor's comment #2, this section has been removed; therefore, this comment is no longer applicable. [No change made]

6) Summary Box (Lines 71–90): Methodological concepts (e.g., variable-centered approaches, population-distribution assumptions) are introduced without grounding and appear disconnected.

In line with the Editor's comment #2, this section has been removed; therefore, this comment is no longer applicable. [No change made]

7) Summary Box (Lines 71–90): The reference to population distribution is unnecessary in this context.

In line with the Editor's comment #2, this section has been removed; therefore, this comment is no longer applicable. [No change made]

Background:

8) Introduction: The rationale for "distinct profiles of compositional movements" is unclear; explain why these profiles represent meaningful archetypes.

We have strengthened the rationale as follows:

In contrast, person-centred approaches such as latent profile analysis allow identification of multiple behavioural profiles within a population and capture meaningful differences in both their movement compositions and consequent health outcomes[14]. Integrating CoDA with latent profile analysis is therefore essential to accurately capture and characterise real-world movement behaviour patterns and their health implications in children. [Page 5, Lines 149-154]

9) Lines 94–115 cover an age range of 5–17 years, which is inconsistent with the study focus of 7–11 years; clarify this early.

The evidence cited in this section is drawn from systematic reviews that were conducted to inform movement behaviour guidelines that are defined for children and adolescents aged 5–17 years. There are no guideline-specific recommendations or pooled evidence available exclusively for the narrower 7-11-year age range included in our study; therefore, the 5–17-year framework represents the most appropriate and consistent context for the study population. [No change made]

10) Lines 104–112 repeat similar points on sedentary behavior; merge these statements.

We have rewritten this paragraph and removed repetition. Our condensed, cleaner paragraph is presented here:

Movement behaviour guidelines recognise the importance of sufficient physical activity across a range of intensities and limiting sedentary behaviour for children's health[6]. However, waking movement behaviours can be accumulated in many ways. However, waking movement behaviours can be accumulated in different ways. These movement behaviour patterns reflect the temporal structure of physical activity and sedentary behaviour during waking hours and are commonly operationalised as activity and sedentary bouts[7]. Evidence regarding which patterns are most beneficial for health remains inconsistent (e.g., in children aged 7-13-year olds [8, 9] and 6-19-year olds [10]), and existing research is largely cross-sectional[1, 2, 11], limiting understanding of the longer-term health implications of suboptimal activity patterns in childhood. Longitudinal studies that move beyond total volumes to examine activity patterns are therefore needed to better inform interventions and policy aimed at improving children's health and wellbeing. [Page 5, Lines 128-140]

11) Lines 119–123 and 124–129 duplicate explanations of variable vs. person-centered approaches; combine for clarity.

We have rewritten this paragraph and merged duplicate explanations as follows:

It is important to move beyond analyses of individual waking movement behaviours towards integrated approaches such as compositional data analysis (CoDA), which accounts for the co-dependency of movement behaviours and enables simultaneous consideration of all activity intensities across the day. Previous CoDA studies have linked school-aged children's movement behaviour compositions adiposity [12, 13] and health-related quality of life (HRQoL)[11]. However, most existing work has applied CoDA within variable-centred approaches (e.g., time reallocation regression models) that assume population homogeneity, thereby overlooking distinct subgroups with different activity accumulation patterns. In contrast, person-centred approaches such as latent profile analysis allow identification of multiple behavioural profiles within a population and capture meaningful differences in both their movement compositions and consequent health outcomes[14]. Integrating CoDA with

latent profile analysis is therefore essential to accurately capture and characterise real-world movement behaviour patterns and their health implications in children. [Page 5, Lines 141-154]

12) Lines 131–135 present the key methodological gap (bout-duration analysis); consider placing this earlier in the introduction.

Thank you for this suggestion. As part of the substantial restructuring of the Introduction following the reviewers' feedback, the flow and coherence of the introduction have improved. As a result, the methodological gaps are now introduced in a logical sequence that builds from existing evidence and analytical approaches, and we therefore chose to retain the current placement. [No change made]

Methods:

13) Lines 173–175: Missing anthropometry due to COVID-19 is noted but not explained; clarify its extent and potential impact.

We have added further information in the methods section:

This led to missing anthropometry data at follow-up for a subset of the cohort, reducing the available sample size for longitudinal analyses of adiposity outcomes and potentially limiting statistical power and representativeness. [Page 7, Lines 201-203]

And in the discussion:

It is important to note that COVID-19 impacted the TransformUs trial significantly. Anthropometric data were not collected for a subset of participants at follow-up due to COVID-19–related disruptions, resulting in missing adiposity outcomes for a subset of participants. This reduced the sample size for longitudinal adiposity analyses and may have limited statistical power and representativeness, with the potential for selection bias if children with missing follow-up measurements differed systematically from those with complete data [46]. Moreover, the study's HRQoL measures were taken during the COVID-19 pandemic, and it is possible that this influenced children's responses, particularly if the timing of data collection coincided with periods of lockdown or school closures, which could have impacted both physical activity patterns and perceptions of health and wellbeing. [Page 19-20, Lines 541-551]

14) Lines 178–204: Accelerometer-processing details are excessive for the main text; move most to supplementary material.

We have moved this information to a new supplementary table. [Supplementary File 2, Table S2]

15) Lines 260–267 combine CoDA transformations, bout metrics, and latent-profile variables; separate these elements to improve readability.

Thank you for this great suggestion. We created separate sections for "Creating movement behaviour compositions with CoDA", "Latent profile analysis" and "Descriptive statistics of profiles and associations with health outcomes". [Statistical analysis]

Model Selection and Profiles:

16) Lines 327–341: Explain the meaning of the covariance structures (VVV vs. EEE) and why VVV was selected.

We have added more information about this in the methods section:

Four latent profile analysis model variants (EEI [equal volume, equal shape and undefined orientation], EEE [equal volume, equal shape, and equal orientation], VVI [varying volume, varying shape and undefined orientation], and VVV [varying volume, varying shape, varying orientation] were considered [32, 33]. The model variants differ in the extent to which they constrain or allow variance and covariance parameters within and between latent profiles [32, 33]. For example, the EEE structure constrains profiles to have equal variances and covariance patterns, such that profiles differ mainly in their mean values, whereas the VVV structure allows variances and covariances to vary freely across profiles, permitting differences in both central tendency and dispersion. [Page 9, Lines 291-299]

17) Lines 342–354 rely heavily on metaphors; provide quantitative contrasts (e.g., effect sizes, standardized values).

Please see our response to reviewer comment #3.

18) Table 3 mixes compositional and arithmetic means without labelling; this should be clarified.

We have now added labels in the footnotes. [Table S5, Supplementary File 2]

Results:

19) Lines 377–386: The statement of "no further associations" overlooks wide confidence intervals; acknowledge this uncertainty.

We agree that confidence intervals provide important information on the precision of effect estimates. However, in our opinion, our results in Table 2 do not include wide confidence intervals for the statistically significant associations; they excluded the null, and are consistent with effect sizes typically observed in early childhood adiposity research. As expected, non-significant associations were characterised by confidence intervals that crossed zero, reflecting greater uncertainty in the estimated effects. Could the reviewer please clarify their comment (i.e., indicate which confidence intervals are considered wide) so that we can address this if necessary?

Discussion:

20) Lines 418–424 repeat novelty statements already covered in the introduction; condense to avoid redundancy.

We have removed this information as suggested by the reviewer.

21) Lines 433–439 offer speculative explanations for adiposity patterns; revise to present these more cautiously or support with evidence.

We have rewritten these statements more carefully and included a reference to a systematic review:

One possible explanation is that replacing sedentary time with LPA may confer relatively smaller benefits for adiposity, as seen in a previous meta-analysis in youth[40]. Nevertheless, longer-term longitudinal studies are needed to better understand the potential health implications of different movement behaviour profiles. [Page 18, Lines 489-493]

22) Lines 441–452 attribute differences to gender without analysis; test gender as a modifier or frame this clearly as speculation.

We have clearly framed this as speculation as follows:

Another potential explanation for these unexpected findings could be gender differences, as the high sedentary-long sedentary bout profile contained more girls than the high sedentary-shorter bouts profile; however, gender was not formally tested as an effect modifier in the present analyses, and this interpretation should therefore be considered exploratory. [Page 19, Lines 505-509]

Research and Policy Implications:

23) Lines 455–465 remain broad; include two concrete examples relevant to school settings.

We have added two specific examples to this section:

Specific promising school-based initiatives include incorporating regular classroom activity breaks to interrupt prolonged sitting [44] and providing equipment during recess to increase engagement in MVPA [45]. These initiatives should be further co-designed with children, parents, and teachers to ensure feasibility, acceptability, and sustained uptake. [Page 19, Lines 525-529]

Limitations:

24) Lines 469–475 mention possible bias from missing data but do not quantify it; provide a comparison of included vs. excluded participants.

We conducted comparisons between participants included in the analytical sample and those excluded prior to analysis and found no meaningful differences in key sociodemographic or baseline characteristics. Given the extensive number of results already presented in the manuscript, we chose not to include these comparisons in the manuscript. Instead, we have now acknowledged this issue in the Limitations section, noting that while included and excluded participants were broadly comparable, some degree of selection bias cannot be entirely ruled out:

Whilst the participants included in the analytical sample did not differ meaningfully from those excluded with respect to their key sociodemographic and baseline characteristics (data not presented), this may have led to some bias in the results. [Page 19, Lines 536-539]

25) Lines 480–481 discuss ceiling effects in HRQoL but do not mention analytic alternatives; state whether Rasch modelling was considered.

We agree that ceiling effects in the HRQoL variables warrant careful consideration. To address this, we used Tobit regression models (described in section "Descriptive statistics of profiles and associations with health outcomes", so not Rasch modelling), which are commonly applied to HRQoL outcomes with censoring at the upper limit. However, the fit did not improve significantly when compared to a standard linear mixed model. Thus, the linear mixed model was conducted. To acknowledge that we did not consider Rasch modelling we have now included the following statement in the discussion:

Future studies could consider applying Rasch modelling to HRQoL measures to better account for ceiling effects. [Page 20, Lines 554-555]

26) Lines 486–489 call for complete 24-hour protocols without linking this to CoDA; clarify why incorporating sleep is essential for 24-hour compositional modelling.

We have included this as follows:

Further research including 24-hour wear protocols, to also incorporate sleep as an important part of the day, is needed to refine these recommendations and explore the long-term effects of different movement patterns on children's health. [Page 20, Lines 563-566]

Reviewer 2 Dr. Ruth Salway, University of Bristol

27) This is an interesting and well-written paper that looks at associations between activity profiles and adiposity and quality of life in children. I think this could be a valuable addition to the evidence, but I have a few issues that need to be addressed, mainly around about some of the analysis and the discussion.

Thank you for your positive comments. We have addressed the issues raised in the below.

28) I don't believe that it is appropriate to include intervention schools at follow-up. Even though this trial was not 'effective' overall, one of the arguments for using latent profile analysis rather than simple PA estimates is that it allows exploration of more complex patterns of activity, and the fact that the programme has previously been found to have an impact on adiposity, sedentary and PA levels makes it extremely likely that it may have impacted these patterns in some way.

Although there was no evidence that the intervention impacted total physical activity and sedentary behaviour, we do agree with the reviewer that inclusion of intervention schools at follow-up may impact some of the findings. To address this concern, we conducted a sensitivity analysis for the longitudinal models only excluding participants from intervention schools at follow-up. The pattern of associations between the latent profiles and the health outcomes were unchanged, and the overall conclusions remained the same. We have now noted this sensitivity analysis in the methods and results sections of the manuscript:

In addition, a separate sensitivity analysis for longitudinal models only was conducted excluding participants from intervention schools at follow-up. [Page 10, Lines 354-356]

Sensitivity analysis excluding children identified as underweight based on their zBMI scores or those in the intervention schools did not change the results. [Page 15, Lines 433-435]

29) The bulk of the follow-up data were collected during the COVID pandemic. There has been a lot of evidence that the pandemic affected PA/SB levels and patterns, adiposity and health and well-being, so any 12-month associations are only valid for during the pandemic and cannot be generalised outside of that time. This requires more justification and far more careful interpretation in the discussion, rather than just a brief mention in the limitations.

This was also raised by reviewer #1 and we have addressed this in our response to comments #15 and #33.

30) The framing of this study as compositional data analysis is not really justified as it doesn't allow the key feature of CoDA which is to explore the potential impact of reallocation of time from one activity to another. This is simply LPA using combinations of movement behaviours across the day – this is useful but it's not CoDA.

We respectfully disagree with Reviewer 2 that the present study does not constitute compositional data analysis. Compositional data analysis is defined by the analysis of data representing parts of a whole that carry relative information and are constrained to sum to a constant, and its defining feature is the use of log-ratio transformations to appropriately model this relative structure. In this study, absolute time-use data were explicitly transformed into log-ratio coordinates prior to modelling, thereby accounting for the co-dependent and relative nature of movement behaviours across the day.

The use of time-reallocation analyses represents one common application of CoDA, but it is not the only way that CoDA can be used. Our study was particularly novel as it first applied

CoDA transformations to the data, before then combining it with latent profile analysis to identify patterns of relative movement behaviour composition. This approach extends CoDA beyond reallocation modelling and represents a novel integration of these methods. The following two papers may be helpful in further explaining the principle of coda: https://www.mdpi.com/1660-4601/17/7/2220 and showcasing that different methods can be applied (not just isotemporal reallocations): https://link.springer.com/article/10.1186/s44167-024-00062-8#Sec7. [No change made]

31) The discussion is under-developed and mainly repeats the results, and there is a lack of reference to the wider literature (four references in total, one of which is just to the PA guidelines). This should be expanded. For example: the COVID impact on followup as in point 2; the fact that half the children are in the lazing lions profile and so any associations may represent the 'typical' child rather than activity patterns; the role of bouts in the profiles; discussion of gender and SES differences; the fact that hummingbirds are nearly all from one geographical area, suggesting there may be issues around generalisability of the results.

Thank you for these great suggestions. We have incorporated these into the discussion and believe the discussion is now more in-depth (and includes several more references). See some examples below:

The high sedentary-long sedentary bout and the high sedentary-shorter bouts profiles were similar in their high levels of daily sedentary behaviour and low levels of physical activity across intensities, however, only the high sedentary-shorter bouts profile reported lower HRQoL compared to the reference high sedentary-long sedentary bout group. It has to be noted that this profile comprised approximately half of the sample, suggesting that observed associations may reflect characteristics of the typical children in this age group [41] rather than effects attributable solely to their movement patterns. However, it may suggest that the way sedentary behaviour is accumulated, specifically, the mean duration of sedentary bouts, may have differential effects on health. [Page 18, Lines 494-502]

In contrast, SES was broadly comparable across profiles (ranging between 48-63%), suggesting that SES is unlikely to account for the observed differences in health outcomes, despite its well-established association with physical activity [43]. [Page 19, Lines 510-513]

Thirdly, the low sedentary-high LPA profile was a relatively small profile compared to the other profiles and included mostly participants from the control group, which may limit the generalizability. [Page 19, Lines 539-541]

It is important to note that COVID-19 impacted the TransformUs trial significantly. Anthropometric data were not collected for a subset of participants at follow-up due to COVID-19–related disruptions, resulting in missing adiposity outcomes for a subset of participants. This reduced the sample size for longitudinal adiposity analyses and may have limited statistical power and representativeness, with the potential for selection bias if children with missing follow-up measurements differed systematically from those with complete data [46]. [Page 19-20, Lines 541-547]

32) The implications suggest that interventions should focus on moving children from one activity profile to another. Firstly, this implies a causal direction to the association (and evidence suggests this is bi-directional with a stronger relationship for adiposity leading to lower PA). But secondly, the suggestions – encouraging children to be active throughout the day without prolonged sitting time – are fairly generic and don't really address how you might facilitate this change and how it might differ between bears and lions, for example.

This was also mentioned by reviewer #1 and we have addressed this in by adding some specific examples to the implications section:

Specific promising school-based initiatives include incorporating regular classroom activity breaks to interrupt prolonged sitting [44] and providing equipment during recess to increase engagement in MVPA [45]. These initiatives should be further co-designed with children, parents, and teachers to ensure feasibility, acceptability, and sustained uptake. [Page 19, Lines 525-529]

Statistical analysis:

33) There are also several issues around the reporting of the statistical analyses: More detail is needed in the main text on the latent profile analysis, rather than hidden in the supplementary materials. For example, VVV, EEI etc are referred to in the results but not explained anywhere; how the optimal number of profiles was chosen. What R package was used?

We have moved the information about the latent profile analysis in the main part of the manuscript and further explained the model variants as follows:

Four latent profile analysis model variants (EEI [equal volume, equal shape and undefined orientation], EEE [equal volume, equal shape, and equal orientation], VVI [varying volume, varying shape and undefined orientation], and VVV [varying volume, varying shape, varying orientation] were considered [32, 33]. The model variants differ in the extent to which they constrain or allow variance and covariance parameters within and between latent profiles [32, 33]. For example, the EEE structure constrains profiles to have equal variances and covariance patterns, such that profiles differ mainly in their mean values, whereas the VVV structure allows variances and covariances to vary freely across profiles, permitting differences in both central tendency and dispersion. [Page 9, Lines 291-299]

The statistical section now also includes mention of the R-package:

Statistical analyses were conducted in RStudio Version 2023.06.2 (R version 4.3.1), using mclust::Mclust() function for the latent profile analysis. [Page 8, Line 268-269]

34) LPA is a probability-based approach. I assume that children have been assigned to the most likely profile for the subsequent analysis; this should be made clear, alongside the limitations of this approach. As the variability is not being accounted for in the

subsequent models, this also suggests that caution should be taken in interpreting borderline p-values. Using ‘most likely profile’ rather than feeding through the variability should also be acknowledged in the limitations.

We have specifically clarified this in the statistical section:

Once the optimal number of latent profiles was selected, the participants were assigned to their most likely profile, created profiles were described, labelled, and used in further regression analyses as a categorical predictor in the subsequent linear models. [Page 10, Lines 322-325]

And in the discussion:

Future studies could consider applying Rasch modelling to HRQoL measures to better account for ceiling effects. Lastly, as latent profile memberships were based on most likely class assignment, classification uncertainty was not accounted for into subsequent models. [Page 20, Lines 554-557]

35) Line 291 re Tobit model: model choice should based on validity of model assumptions and not on model fit – it doesn’t matter how good the fit is of a linear model if it breaks the underlying assumptions because this invalidates any results.

We agree that model choice should be guided by the validity of underlying assumptions rather than goodness-of-fit alone. In the present analyses, we explicitly assessed the assumptions of the selected linear mixed-effects models, including residual normality, homoscedasticity, and linearity, and found no meaningful violations. Diagnostic plots indicated that model residuals were approximately normally distributed and showed no evidence of heteroscedasticity, supporting the validity of the linear model assumptions (see below, not presented in manuscript).

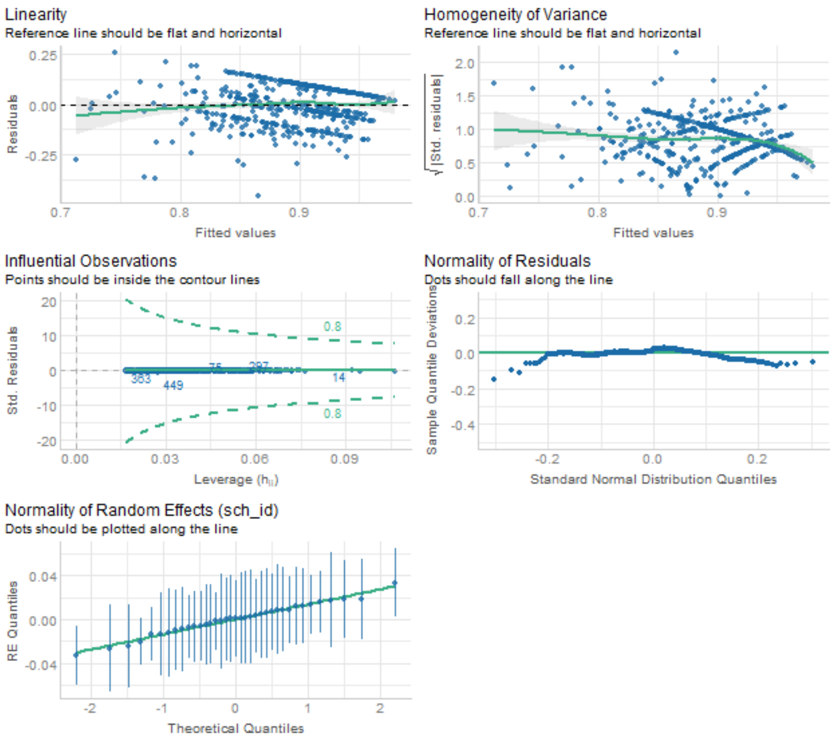

We also explored alternative modelling approaches appropriate for censored outcomes, including mixed-effects Tobit models treating HRQoL scores as censored at the upper bound. These models yielded qualitatively similar results to the linear mixed-effects models, but were more complex to implement in the presence of random effects and did not materially alter inference. Given that the assumptions of the linear models were met and results were robust across modelling approaches, we retained the linear mixed-effects models for clarity and interpretability. [No changes made]

36) The term ‘statistical significance’ should be avoided, and instead report estimates and confidence intervals alongside p-values.

We have removed this term throughout the manuscript and presented 95% confidence intervals where needed.

37) There are a lot of p-values reported, and so care should be taken on identifying associations based on borderline p-values. Also analysing the subdomains of the HRQoL feels like fishing for ‘significant’ associations, especially when there is no evidence of association with HRQoL overall.

Whilst we decided to analyse the individual HRQoL domains a-priori, we have now removed these from manuscript. In addition, we have presented 95% confidence intervals where needed.

38) In Tables 4 & 5, the profiles are mutually exclusive and so are a categorical variable and so the relevant p-value should be for a Wald test for the category as a whole, and not separate p-values for each profile.

We have included the Wald test results in the results sections as follows:

However, the omnibus Wald test for differences across profiles indicated uncertainty in the overall profile effect ($\chi^2(3) = 4.68$, $p = 0.20$), zWC ($\chi^2(3) = 7.02$, $p = 0.07$). [Page 15, Lines 426-427]

Wald test for longitudinal associations showed no evidence of a longitudinal profile effect across outcomes. [Page 15, 432-433]

Minor points:

39) It's not always clear in the introduction which evidence refers to children and young people generally and which relates to the specific age group 7-11 explored in this study.

We have added the age groups throughout the introduction to clarify.

40) Line 164: Years 3 and 4 (aged 7-11 years) - aren't these ages 8-10?

Whilst children are typically 8-10-years old in grades 3 and 4, there were some 7- and 11-year-olds in our sample.

41) Line 172: presumably measurements were not the same – do you mean measurement protocols (eg days of wear etc) as opposed to data collection protocols?

Apologies, we meant protocols and have now added this as follows:

All measurement protocols were identical at both time-points, however… [Page 6, Line 197]

42) Line 180+ are these wrist or hip worn accelerometers?

Apologies, we forgot to include this information. We have now added:

Of the 1,370 consenting students, 1,296 (94.6%) participated in baseline data collection, of whom 1,197 (92.4%) were provided with an accelerometer to wear on their right hip for a period of eight days. [Page 7, Lines 207-209]

43) ln 349 – hummingbirds nearly all from NSW – this does not suggest that these profiles can be interpreted as generalisable across populations then

See our response to reviewer comment #33.

44) ln 347 – I'm not sure two months younger on average is a difference worth noting.

The difference between the Low sedentary-high LPA (8.8) and the High sedentary-long sedentary bout (9.2) groups was on average about four months, which we felt like was worth mentioning, considering we know that movement behaviours change with age.

45) Table 2: please add the fit indicators for more profiles, so it is clear why 4 profiles is to be preferred to more.

All fit indicators (for 1-9 profile solutions) can be found in Figure S1.

46) Line 378: surely the lazing lions as the largest profile (so representing the most typical activity patterns) would make a better reference category? Also 'referent' is the wrong word; should be 'reference'.

The author team carefully considered several options for the reference category and ultimately selected the high sedentary–long sedentary bout profile, as it was considered the least favourable movement profile. We have changed the terminology to 'reference' instead of 'referent' throughout.

47) Tables 4 & 5: it's not clear how to interpret the magnitude of these coefficients – is a difference of 0.27 in zBMI meaningful? Also is perceived health the same as the VAS scale? If so, can you define somewhere/be consistent.

We have given this some more context in the discussion:

Compared to the high sedentary-long sedentary bout profile, the high MVPA-short sedentary bout profile had more favourable adiposity levels cross-sectionally (−0.27 zBMI units; 95% CI −0.51 to −0.02). Although this estimate should be interpreted in the context of limited evidence for overall differences across profiles, the observed difference of approximately 0.27 zBMI units represents around one quarter of a standard deviation (SD) and may be clinically meaningful, given evidence that each 1 SD increase in childhood BMI z-score is associated with an approximately 30% higher risk of cancer in adulthood [39]. [Page 18, Lines 468-474]

48) Line 430-32: 'almost complying with physical activity guidelines'. This is not true – it's not meaningful to compare a group (profile) average to an individual threshold, and due to variability quite a lot of children in these profiles will achieve 60+mins. It might be useful to actually calculate the proportion of children in each profile who achieve the guidelines.

We agree that it is better to present the proportions, so we have now also included these in this section:

Both the high MVPA-short sedentary bout profile and the low sedentary-high LPA profile exhibited high MVPA levels compared to other two groups, almost complying with current physical activity guidelines recommending at least 60 minutes of MVPA per day for children (achieving on average 59.6 and 56.2 min/d, on average, with 44% and 30% of participants meeting the guidelines, respectively)[6]. [Page 18, Lines 479-483]

49) Supplementary Table S2: reference 2 is incomplete, and references for BIC etc are needed. If possible, use references to the original methods rather than to tutorials.

We have fixed up these references as requested by the reviewer. We have kept the tutorial as it is a pre-print with DOI.

50) No Supplementary Table S3?

Several changes have been made to the supplementary tables in response to reviewer feedback. As a result, Supplementary Table S3 has been removed. Importantly, we have verified that all supplementary files are included in the current submission.

51) Supplementary Tables S4 & S5: these would be easier to interpret if they reported odds ratios and confidence intervals (and p-values should be explicitly reported rather than using * for statistical significance).

These tables have been removed. See our response to reviewer comment #39.

VERSION 2 - REVIEW

**Reviewer**	**1**
**Name**	**Alkazemi, Dalal**
**Affiliation**	**Kuwait University, Food Science and Nutrition**
**Date**	**28-Jan-2026**
**COI**	

This manuscript addresses an important public health question by combining compositional data analysis with latent profile analysis to characterise children's movement patterns and their associations with adiposity and HRQoL. The methodological approach is sophisticated, and the use of device-based measures with both cross-sectional and longitudinal data is a clear strength. The paper is generally well written and transparent in reporting. That said, several issues limit the interpretability and impact of the findings.

- The key limitation is that omnibus tests across profiles were largely non-significant, both cross-sectionally and longitudinally, yet the discussion places considerable emphasis on pairwise comparisons, particularly for the high MVPA–short sedentary bout profile. This weakens the central narrative. I would recommend reframing the conclusions more cautiously and foregrounding that overall profile effects were uncertain, with only limited exploratory pairwise signals.

- The clinical relevance of the reported cross-sectional zBMI difference (~0.27 SD) feels overstated given the lack of global profile effects and absence of longitudinal associations.

Language around “unique implications for health” should be tempered, avoiding suggestions of meaningful impact without stronger longitudinal support.

- Attrition and missingness at follow-up are substantial, with only around 55% contributing adiposity data. Although COVID-related disruption is acknowledged, the manuscript would benefit from a clearer comparison of baseline characteristics between participants retained and lost to follow-up to better assess potential selection bias.

- Profile membership uncertainty is not accounted for in downstream regression models, as analyses rely on most-likely class assignment. This should be discussed more explicitly as a methodological limitation, given its potential to bias estimates toward significance.

- The low sedentary high LPA profile is small (n=54) and largely drawn from one state, raising concerns about stability and generalizability. Please clarify whether any bootstrapping or robustness checks were undertaken to assess profile reproducibility.

- HRQoL outcomes show strong ceiling effects, which you acknowledge. Given this, conclusions related to HRQoL should be softened further, and it may be worth stating more clearly that EQ-5D-Y may lack sensitivity in generally healthy child populations.

- Please clarify why mean bout durations were entered untransformed despite evident skewness and briefly justify the decision to prioritize interpretability over distributional assumptions. The rationale for selecting the high sedentary long sedentary bout profile as the reference group should also be stated more explicitly in the main text rather than only in tables.

- Gender differences across profiles are described descriptively but not formally explored. Either interaction effects should be tested, or these observations clearly framed as speculative.

- Diet and sleep are noted as limitations, but given their relevance to adiposity and HRQoL, this deserves stronger emphasis when interpreting null longitudinal findings.

- The discussion occasionally implies that certain movement “patterns” support health, yet the longitudinal analyses do not confirm this. I recommend consistently framing findings as baseline associations only. Similarly, policy implications should be scaled back in light of the largely null longitudinal results.

- Consider tightening the abstract conclusions to better reflect statistical uncertainty and adding a brief schematic or summary table of profile characteristics to the main manuscript to aid reader interpretation.

Reviewer 2

Name Salway, Ruth

Affiliation University of Bristol, Centre for Exercise, Nutrition and Health Sciences

Date **06-Feb-2026**

COI

I'd like to congratulate the authors on a detailed and thoughtful response to my review comments. The changes they have made have clarified many of my concerns, and I appreciate the extra sensitivity analysis for the inclusion of intervention schools at follow-up. The discussion in particular has been extensively reworked and now provides a much improved consideration of the implications of the results.

There are a couple of places where we still disagree (!), but the authors argued their point well and I accept these as differences in opinion. They have provided sufficient justification for their thinking, so the reader can make up their own mind.

I would now recommend this paper for publication.

VERSION 2 - AUTHOR RESPONSE

<u>Reviewer 1 Dr. Dalal Alkazemi, Kuwait University:</u>

1) This manuscript addresses an important public health question by combining compositional data analysis with latent profile analysis to characterise children's movement patterns and their associations with adiposity and HRQoL. The methodological approach is sophisticated, and the use of device-based measures with both cross-sectional and longitudinal data is a clear strength. The paper is generally well written and transparent in reporting. That said, several issues limit the interpretability and impact of the findings.

Thank you for your positive comments regarding the analysis, design and reporting of the study. We have further revised the manuscript to improve interpretability and impact of the findings using your feedback. We have listed these changes below in the response to your comments 2-12. Thank you.

2) The key limitation is that omnibus tests across profiles were largely non-significant, both cross-sectionally and longitudinally, yet the discussion places considerable emphasis on pairwise comparisons, particularly for the high MVPA–short sedentary bout profile. This weakens the central narrative. I would recommend reframing the conclusions more cautiously and foregrounding that overall profile effects were uncertain, with only limited exploratory pairwise signals.

We agree that the discussion contained a too strongly worded explanation of results, so we have toned this down at several spots in the discussion as follows:

"There was limited evidence for overall differences in health outcomes across profiles, with little evidence from pairwise comparisons. Only the high MVPA-short sedentary bout profile

showed more favourable adiposity levels cross-sectionally (−0.27 zBMI units; 95% CI −0.51 to −0.02) compared to the high sedentary-long sedentary bout profile. Although this estimate should be interpreted in the context of limited evidence for overall differences across profiles, the observed difference of approximately 0.27 zBMI units represents around one quarter of a standard deviation (SD) and may be clinically meaningful, given evidence that each 1 SD increase in childhood BMI z-score is associated with an approximately 30% higher risk of cancer in adulthood [39]. At the 12-month follow-up, only the high sedentary–shorter bouts profile showed lower reported HRQoL compared with the reference high sedentary–long sedentary bout group; however, the confidence interval included the null (95% CI −0.05 to 0.00; p = 0.06) and the omnibus test did not suggest overall profile effects, and this finding should therefore be interpreted with caution." [Page 18, Lines 483-495]

"The pairwise finding (despite the lack of evidence from overall group omnibus test) that the high MVPA-short sedentary bout profile had favourable adiposity markers cross-sectionally than the high sedentary-long sedentary bout profile is consistent with previous research showing that MVPA is most strongly and positively associated with improved health outcomes, compared to lower-intensity activities[2, 40]." [Page 18, Lines 500-505]

"The high sedentary-long sedentary bout and the high sedentary-shorter bouts profiles were similar in their high levels of daily sedentary behaviour and low levels of physical activity across intensities, however, exploratory pairwise comparisons showed that only the high sedentary-shorter bouts profile reported lower HRQoL compared to the reference high sedentary-long sedentary bout group." [Page 18, Lines 512-516]

3) The clinical relevance of the reported cross-sectional zBMI difference (~0.27 SD) feels overstated given the lack of global profile effects and absence of longitudinal associations. Language around "unique implications for health" should be tempered, avoiding suggestions of meaningful impact without stronger longitudinal support.

We have removed the language around "unique implications" and replaced this with tempered statements such as:

"Four distinct movement profiles were identified. Few pairwise differences between health outcomes were observed. While MVPA remains a key factor for promoting healthy body weight, our findings suggest that a variety of movement patterns - including those characterized by lower sedentary time and higher LPA - may support health in children." [Page 20, Lines 583-586]

4) Attrition and missingness at follow-up are substantial, with only around 55% contributing adiposity data. Although COVID-related disruption is acknowledged, the manuscript would benefit from a clearer comparison of baseline characteristics between participants retained and lost to follow-up to better assess potential selection bias.

We have added a comparison of baseline characteristics between those included in the longitudinal analysis versus those that were not. This is described in the methods, results and discussion as follows:

"Participants included in the longitudinal analyses were compared to those with no follow-up data with either a Chi-squared test (categorical variables) or a one-way ANOVA (continuous variables)." [Page 8, Lines 275-277]

"Participants included in the longitudinal analyses were significantly more likely to be from a major city, older and have higher baseline zWC (all p<0.05). No differences were observed for sex, SES, profile membership, or baseline zBMI, QoL or VAS health." [Page 11, Lines 380-383]

"Whilst the participants who provided data at baseline did not differ largely from those in the longitudinal analytical sample, they were less likely to be from a major city, younger and have a lower baseline zWC, which may have led to some bias in the results." [Page 19, Lines 555-558]

5) Profile membership uncertainty is not accounted for in downstream regression models, as analyses rely on most-likely class assignment. This should be discussed more explicitly as a methodological limitation, given its potential to bias estimates toward significance.

We have added the following sentence in the limitations section:

"The downstream analyses were also based on most-likely class assignment and did not account for uncertainty in profile membership, which may have biased estimates toward statistical significance and should be considered when interpreting these findings." [Page 20, Lines 560-563]

6) The low sedentary high LPA profile is small (n=54) and largely drawn from one state, raising concerns about stability and generalizability. Please clarify whether any bootstrapping or robustness checks were undertaken to assess profile reproducibility.

We thank the reviewer for raising this important point. We did not undertake bootstrapping; however, several model evaluation steps were conducted that speak to the reproducibility and stability of the solution.

Model selection followed established criteria: we compared solutions across k = 2–[X] profiles using multiple indicators, selecting the final solution based on fit indices and interpretability of the profile structure. The selected solution demonstrated acceptable classification certainty (entropy = 0.78), suggesting reasonably well-separated profiles.

We acknowledge, however, that the Low Sedentary/High LPA profile (n = 54) is notably small and geographically concentrated, which limits confidence in the generalisability of this profile. We have added explicit acknowledgement of this in the Limitations section, noting

that findings for this profile should be interpreted with caution and that replication in larger, more geographically diverse samples is warranted:

"Thirdly, the low sedentary-high LPA profile was relatively small compared with the other profiles and included mostly participants from the control group, which may limit the generalisability of this profile." [Pages 19-20, Lines 558-560]

7) HRQoL outcomes show strong ceiling effects, which you acknowledge. Given this, conclusions related to HRQoL should be softened further, and it may be worth stating more clearly that EQ-5D-Y may lack sensitivity in generally healthy child populations.

We have added this statement in the discussion:

"Fifthly, it has to be noted that in 'otherwise healthy' general young populations, there is a strong ceiling effect for the 'no problems' level in mobility, independence, and daily living activities. The EQ-5D-Y may lack sensitivity in generally healthy child populations and therefore may have had limited ability to detect associations in the current study." [Page 20, Lines 574-577]

8) Please clarify why mean bout durations were entered untransformed despite evident skewness and briefly justify the decision to prioritize interpretability over distributional assumptions.

This is described in the results section as follows:

"Since the log-transformed bout duration did not yield better model probabilities, the original four-profile model was adopted due to easier interpretability." [Page 14, Lines 408-410]

9) The rationale for selecting the high sedentary long sedentary bout profile as the reference group should also be stated more explicitly in the main text rather than only in tables.

The rationale for selecting this group as the reference profile is listed in the results section:

"The high sedentary-long sedentary bout profile was selected as the reference profile, as they were considered the least favourable movement profile (Supplementary File 2, Table S5)." [Page 15, Lines 437-439]

10) Gender differences across profiles are described descriptively but not formally explored. Either interaction effects should be tested, or these observations clearly framed as speculative.

Table S5 (in the supplementary files) displays the formal testing results for group comparisons (Chi-squared), including those for gender. We did however acknowledge that we did not include gender as a potential effect modifier in the discussion:

"Another potential explanation for these unexpected findings could be gender differences, as the high sedentary-long sedentary bout profile contained more girls than the high sedentary-shorter bouts profile; however, gender was not formally tested as an effect modifier alongside the group comparisons presented in the present analyses, and this interpretation should therefore be considered exploratory." [Page 19, Lines 523-528]

11) Diet and sleep are noted as limitations, but given their relevance to adiposity and HRQoL, this deserves stronger emphasis when interpreting null longitudinal findings.

We have clearly included the role of diet and sleep for long term health specifically in the limitations section:

"A number of limitations should be considered when interpreting these findings. Firstly, this study did not account for sleep behaviours and diet, which are known to influence both physical activity patterns and health outcomes long-term in children [6]." [Page 19, Lines 551-553]

12) The discussion occasionally implies that certain movement "patterns" support health, yet the longitudinal analyses do not confirm this. I recommend consistently framing findings as baseline associations only. Similarly, policy implications should be scaled back in light of the largely null longitudinal results.

We have altered the research and policy implications and removed the text that heavily focused on long-term health outcomes. Rather we know discuss the diversity in movement patterns and that interventions should consider this, since there was some cross-sectional evidence to suggest this may have potential. We have used more careful language in doing so:

"The findings from this study underscore the importance of recognizing the diversity in children's movement behaviour patterns when developing health interventions and policies. The identification of distinct profiles impacts how interventions should be tailored to address these specific movement behaviours and consider their activity patterns, rather than only aiming to increase overall activity levels. An example of how this could be done is by providing various opportunities for children to be active throughout the day, such as through whole-school movement initiatives, encouraging children to reach sufficient MVPA throughout the day with limited prolonged sitting - aiming for a replication of the high MVPA-short sedentary bout profile's behaviour which was associated with best adiposity outcomes, albeit cross-sectionally. Specific promising school-based initiatives include incorporating regular classroom activity breaks to interrupt prolonged sitting [44] and providing equipment during recess to increase engagement in MVPA [45]. These initiatives should be further co-designed with children, parents, and teachers to ensure feasibility, acceptability, and sustained uptake." [Page 19, Lines 535-548]

13) Consider tightening the abstract conclusions to better reflect statistical uncertainty and adding a brief schematic or summary table of profile characteristics to the main manuscript to aid reader interpretation.

We have changed the abstract conclusions and they now no longer refer to "unique impacts for health":

"Conclusions: Four distinct movement profiles were identified. Few pairwise differences between health outcomes were observed. While MVPA remains a key factor for promoting healthy body weight, our findings suggest that a variety of movement patterns - including those characterized by lower sedentary time and higher LPA - may also support health in children." [Page 3, Lines 74-78]

The table with profile characteristics can be found in the supplementary files, Table S5. We had initially presented this in the main manuscript, however, the BMJ Open Editorial Office suggested in our last revision to place this into the supplementary files due to it size. [No change made]

Reviewer 2 Dr. Ruth Salway, University of Bristol

14) I'd like to congratulate the authors on a detailed and thoughtful response to my review comments. The changes they have made have clarified many of my concerns, and I appreciate the extra sensitivity analysis for the inclusion of intervention schools at follow-up. The discussion in particular has been extensively reworked and now provides a much improved consideration of the implications of the results. There are a couple of places where we still disagree (!), but the authors argued their point well and I accept these as differences in opinion. They have provided sufficient justification for their thinking, so the reader can make up their own mind. I would now recommend this paper for publication.
